# Bacterial community function increases leaf growth in a pitcher plant experimental system

**DOI:** 10.1128/msystems.01298-24

**Published:** 2024-11-25

**Authors:** Jessica R. Bernardin, Erica B. Young, Sarah M. Gray, Leonora S. Bittleston

**Affiliations:** 1 Department of Biological Sciences, Boise State University, Boise, Idaho, USA; 2 Department of Biological Sciences and School of Freshwater Sciences, University of Wisconsin-Milwaukee, Milwaukee, Wisconsin, USA; 3 Department of Biology-Ecology and Evolution, University of Fribourg, Fribourg, Switzerland; University of Hawaiʻi at Mānoa, Honolulu, Hawaii, USA

**Keywords:** microbial community function, metatranscriptomics, phyllosphere, microbe-plant interactions, host health, metagenomics, pitcher plant, *Sarracenia purpurea*

## Abstract

Across diverse ecosystems, bacteria and their hosts engage in complex relationships having negative, neutral, or positive interactions. However, the specific effects of leaf-associated bacterial community functions on plant growth are poorly understood. Although microbes can promote plant growth through various biochemical mechanisms, investigating the community’s functional contributions to plant growth remains to be explored. To address this gap, we characterized the relationships between bacterial community function and host plant growth in the purple pitcher plant (*Sarracenia purpurea*). The main aim of our research was to investigate how different bacterial community functions affect the growth and nutrient content in the plant. Previous research has suggested that microbial communities aid in prey decomposition and subsequent nutrient acquisition in carnivorous plants, including *S. purpurea*. However, the specific functional roles of bacterial communities in plant growth and nutrient uptake are not well known. In this study, sterile, freshly opened pitchers were inoculated with three functionally distinct, pre-assembled bacterial communities. Bacterial community composition and function were measured over 8 weeks using physiological assays, metagenomics, and metatranscriptomics. Distinct community functions affected plant traits; a bacterial community enriched in decomposition was associated with larger leaves with almost double the biomass of control pitchers. Physiological differences in bacterial communities were supported by metatranscriptomics; for example, the bacterial community with the highest chitinase activity had greater expression of transcripts associated with chitinase enzymes. The relationship between bacterial community function and plant growth observed here indicates potential mechanisms, such as chitinase activity, for host-associated bacterial functions to support pitcher plant growth.

**IMPORTANCE:**

This study addresses a gap in understanding the relationships between bacterial community function and plant growth. We inoculated sterile, freshly opened pitcher plant leaves with three functionally distinct bacterial communities to uncover potential mechanisms through which bacterial functions support plant health and growth. Our findings demonstrate that distinct community functions significantly influence plant traits, with some bacterial communities supporting more plant growth than in control pitchers. These results highlight the ecological roles of microbial communities in plants and thus ecosystems and suggest that nutrient cycling is an important pathway through which microbes support host plant health. This research provides valuable insights into plant-microbe interactions and the effects of diverse microbial community functions.

## INTRODUCTION

Microbiomes have strong effects on the health and fitness of their hosts (1, 2). Host plant growth and physiology are influenced by the presence of leaf-associated (phyllosphere) microbes, with microbial taxa acting as mutualists, commensals, and antagonistic pathogens (3–6). In the case of mutualistic plant microbial interactions, the reciprocal benefits between organisms may include phyllosphere microbes contributing to nutrient acquisition, photosynthetic efficiency, stress tolerance, or pathogen defense, while in return, the microbes receive habitat and resources that support their growth and reproduction (7–9). Phyllosphere microbes exist in complex microecosystems (10, 11), where they interact with each other and their host in dynamic ways. Understanding these relationships is an important first step in using microbial communities as a tool to improve plant growth.

Microbial functions have significant impacts on plant health and productivity. For example, the addition of synthetic phyllosphere communities was shown to increase fruit production in greenhouse-grown tomatoes (6) and increase leaf sugar content and metabolic diversity in field-grown *Agave tequilana* (12). Plants reap many benefits from hosting microbial communities (4, 11). Nutrient cycling interactions between microbes and plants can occur directly, for example, via bacterial nitrogen fixation, and indirectly, for example, via increased bacterial growth that influences host plant metabolism (4, 13). Many bacteria associated with plant-microbe interactions also have pathways to produce phytohormones, like auxin or cytokinins, which support plant cell metabolism and growth (4, 14–17). Plant growth-promoting bacteria can also mediate host stress responses, including increasing drought tolerance, thermotolerance, and UV protection (4, 18, 19). The microbes in these interactions benefit from specialized niches that offer resources, habitat, and protection from environmental extremes (8). Despite these demonstrated host-microbe beneficial interactions, research directly connecting host physiological responses with bacterial community function remains limited. Here, we define bacterial community function as the collective metabolic processes of all the individual bacteria within a community. We addressed this knowledge gap by examining both bacterial and plant physiology, using metabarcoding, metagenomics, and metatranscriptomics of bacterial communities and measuring plant responses in a pitcher plant model system.

The carnivorous pitcher plant, *Sarracenia purpurea* L., uses water-filled, cup-shaped leaves to trap and digest insects to supplement the acquisition of nutrients from low-nutrient soils (9, 20). These leaves or “pitchers” host an aquatic micro-ecosystem composed of microbes, fungi, protists, and invertebrates (21, 22). Typically, the organisms found in all pitcher aquatic communities are from the same phylogenetic clades with bacterial communities dominated by Microbacteriaceae, Chitinophagaceae, and Comamonadaceae families (23–25). These communities change over time during succession and with biogeography (23, 24). Each pitcher is sterile inside prior to opening (26), and microbes are recruited into the communities from the air, rainwater, and surrounding environments (25, 27). The pitcher plant represents a unique environment, characterized by specialized leaf morphology but we believe this system is well-suited for measuring the impacts of microbial community function on host plant health. The microbes provide critical ecosystem functions to the host plants by mediating insect prey digestion through the production of extracellular hydrolytic enzymes. Likely, the bacterial production of protease and chitinase enzymes that digest protein and chitin-rich insect prey is especially important for this carnivorous plant species (28–30). *Sarracenia purpurea* pitcher plants do not produce chitinase enzymes (28, 31). Thus, they may rely heavily on the bacterial extracellular enzymes to catalyze the decomposition of insect prey and support the mineralization of carbon, nitrogen, and other nutrients for the host plant and the whole micro-ecosystem.

This is an ideal model plant-microbe system to examine bacterial functions in controlled manipulative experiments, serving as an analog for other systems where experimentation is more challenging. Although previous research has hypothesized that pitcher plant leaf-associated bacterial communities benefit the host plant (9, 25, 32), no studies have specifically measured a direct benefit to the plant. Clearly, the pitcher plant represents a unique and unusual environment, characterized by specialized leaf morphology and prey digestion as its primary ecosystem function. Despite this, we believe this system is well-suited for measuring the impacts of community function on host health. The liberation of nutrients by microbes can significantly influence host nutrient acquisition—a common function of plant-associated communities (33, 34). Moreover, we believe that directly characterizing the microbial benefits to the host plant can provide insights relevant to other systems where microbes play a crucial role in nutrient cycling and decomposition.

**Fig 1:**
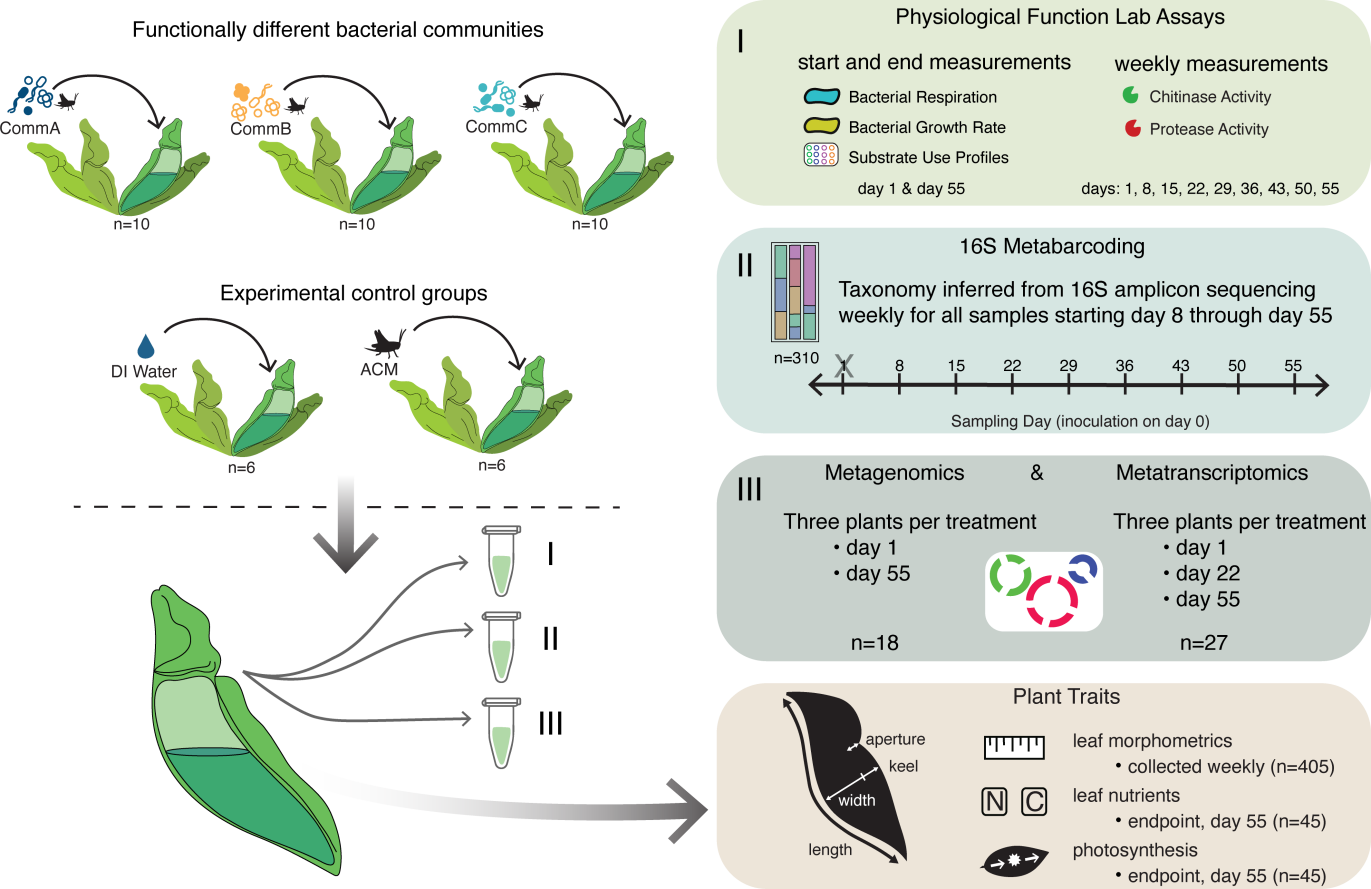
Experimental design. Three functionally distinct bacterial communities (CommA, CommB, and CommC) growing in sterile acidified cricket media (ACM) were inoculated into sterile pitchers and plants incubated in growth chambers for 8 weeks. Sterile deionized (DI) water or ACM was added to additional pitchers as experimental controls. Pitcher fluid samples were collected weekly to characterize bacterial community composition and function: chitinase and protease activity, community respiration, growth rate, organic substrate use profiles, 16S amplicon sequencing, metagenomics, and metatranscriptomics. Plant functional traits were also assessed, including morphometric measurements of pitchers, as well as end-of-experiment measures of leaf biomass, leaf nutrient content, and photosynthetic rate.

To connect the effects of bacterial function to plant growth, we designed a manipulative experiment using pitcher plant leaves and bacterial communities with different physiological functional profiles, measured as different levels of activity across a range of community functions (Fig. S1). These community physiological functions, including hydrolytic enzyme activity, organic substrate use, and respiration rates, were characterized for the three distinct experimental bacterial communities prior to inoculation into host plants, and the effects of these functionally distinct bacterial communities on host plants, were examined over 8 weeks (Fig. 1).

We asked four specific questions:

- Do bacterial communities with different functional profiles have differential effects on plant traits, including leaf growth and nutrient content?
- Are there taxonomic characteristics of these bacterial communities that can explain the observed functional differences?
- Can bacterial functions, measured directly with physiological assays, be linked with differential gene expression (metatranscriptomics) to better understand how bacterial community function impacts their host?
- Are there specific functions, for example, hydrolytic enzyme activity or carbon substrate use, that drive these effects on host plants?

We hypothesized that communities with specific physiological functions (e.g., high chitinase activity) would differentially express genes related to degradation, helping to release and make available more nutrients to enhance host plant growth.

## RESULTS

### Starting communities were functionally different

**Fig 2:**
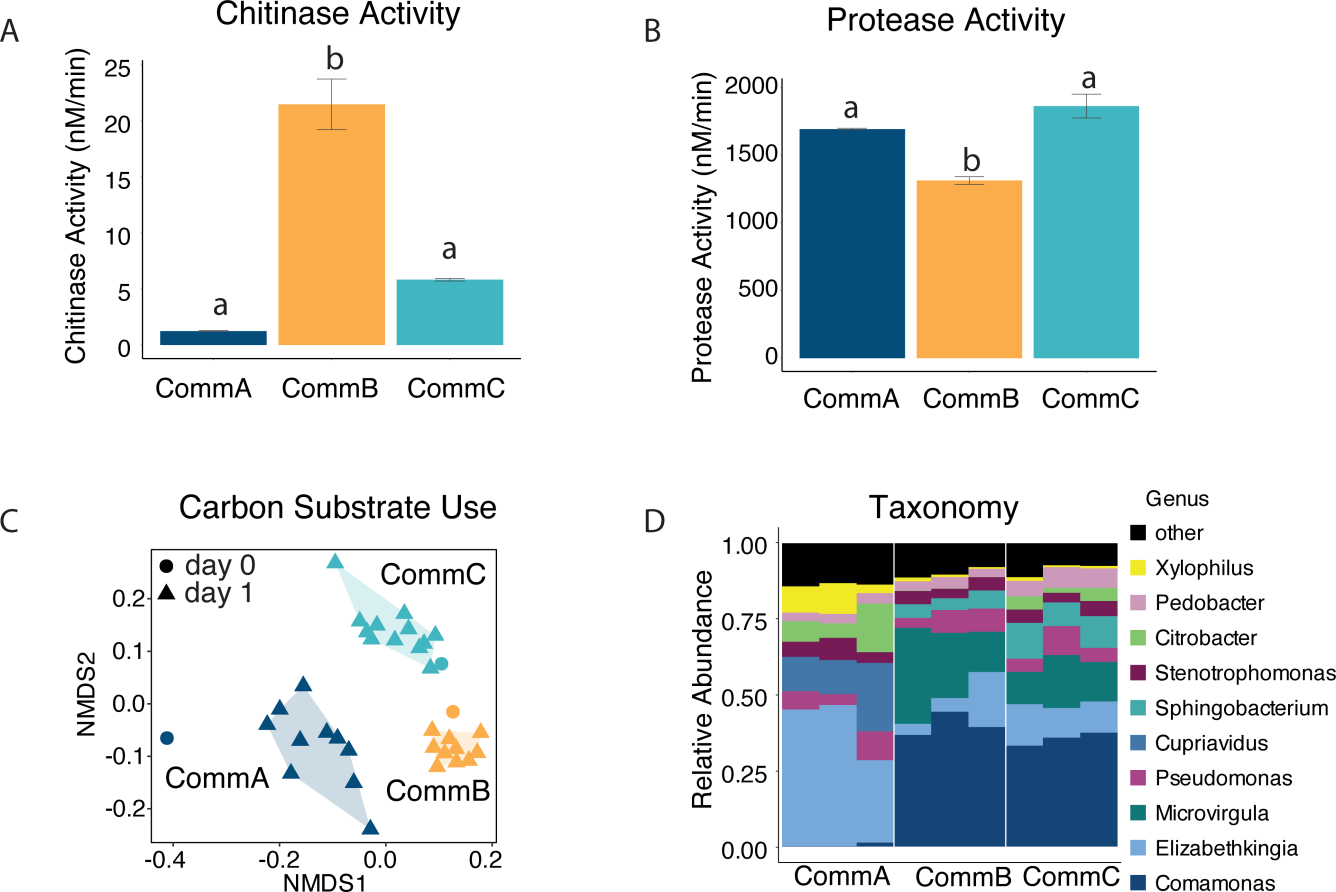
The three bacterial communities had different physiological functions measured before pitcher inoculation. (**A**) Chitinase activity and (**B**) protease activity of starting bacterial communities; letters above bars indicate statistical differences between communities based on a Bayesian GLM. (**C**) A nonmetric multidimensional scaling ordination (k = 2, stress = 0.138) based on Bray-Curtis dissimilarities for carbon substrate use (EcoPlate) for all three treatment communities prior to inoculation (day 0) and 24 hours after inoculation (day 1). (**D**) Relative abundance of the top 10 genera of the three communities 24 hours after inoculation (day 1) based on metagenomic sequencing of three representative pitcher samples in each treatment.

Bacterial communities were established from wild pitcher plant aquatic microbial communities (see Materials and Methods) using acidified cricket media and serial transfers every 72 hours for 63 days prior to being used in this experiment. These communities were functionally characterized in a previous experiment (35) and immediately before being used as microbial inoculum for the current experiment. The three communities (CommA, CommB, and CommC) differed in their chitinase activity, protease activity, bacterial respiration, and/or EcoPlate organic carbon substrate use profiles (Fig. 2; Fig. S2) despite similar bacterial densities. Prior to inoculation, CommB had the highest chitinase activity (mean = 21.5 nM/min, 95% credible intervals [95% CIs] = 17.3, 27.4) and lowest protease activity (mean = 1,315 nM/min, 95% CIs = 1,172, 1,467) (Fig. 2A and B, statistical differences shown in Fig. S2), and bacterial respiration was lower in CommB (mean = 2,632 CO_2_ppm/h, 95% CIs = 2,063, 3,441) than the other two communities (Fig. S2). Using a permutational multivariate analysis of variance (PERMANOVA), we observed strong differences in carbon substrate use profiles among the three bacterial communities, both in the community cultures prior to inoculation (day 0) and when resampled from the pitchers 24 hours after inoculation (day 1) (Fig. 2C; Fig. S3; Table S1). CommB was the most distinct, while CommA and CommC exhibited similar hydrolytic enzyme activities, all three communities had distinct carbon substrate use profiles. We found significant differences in community composition between the treatments on day 1 based on metagenomic sequencing (Fig. 2D; PERMANOVA; *R*^2^ = 0.78, *F*_2,8_=10.382, *P* = 0.005). There were no measured differences in bacterial community growth rates prior to inoculation or at any point during the experiment (mean_CommA_ = 0.69, mean_CommB_ = 0.71, mean_CommC_ = 0.68; Fig. S2).

### Bacterial function affects plant traits

**Fig 3:**
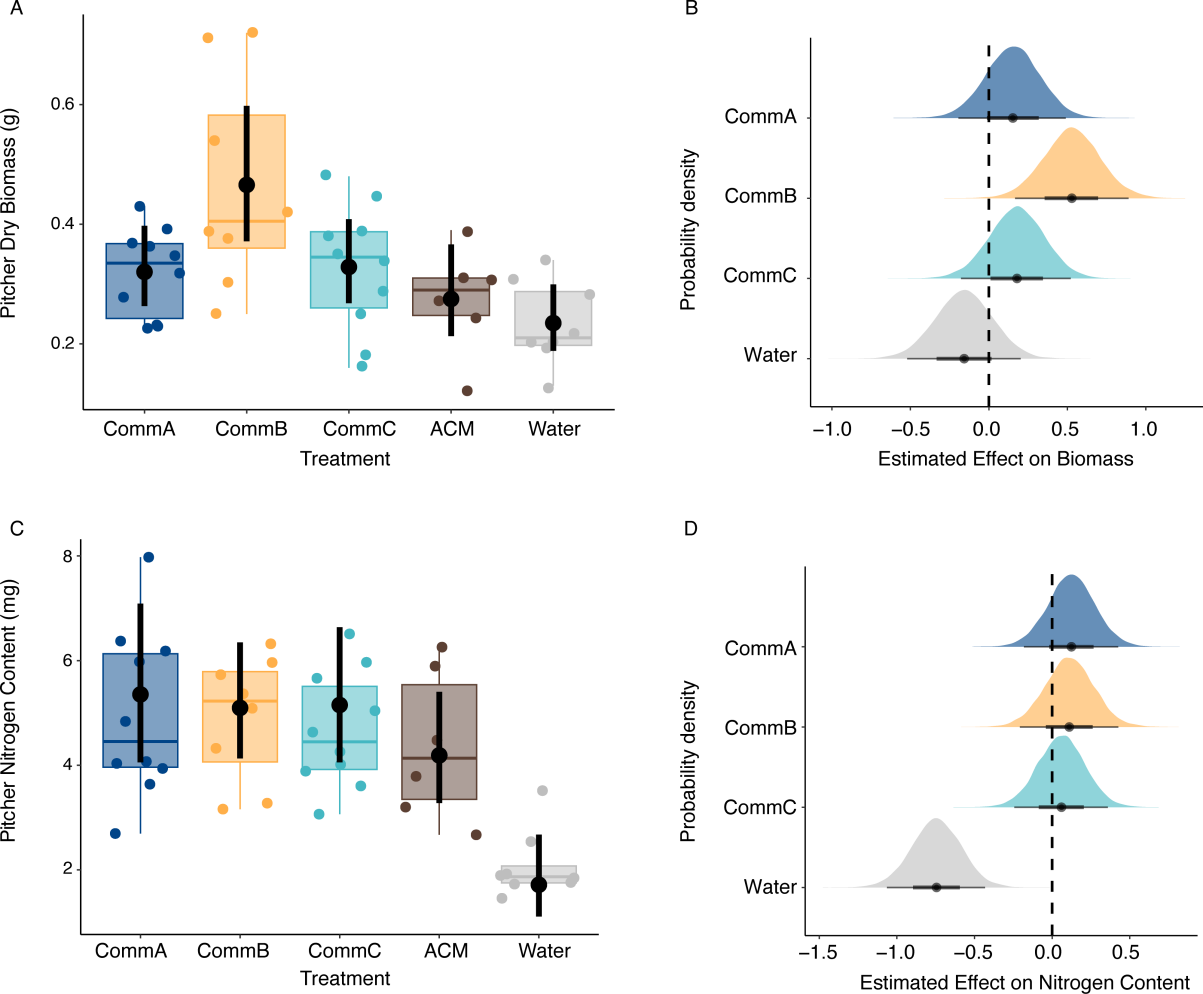
Bacterial community treatment affects plant biomass. (**A**) Bayesian GLMM marginal effects of treatment on target pitcher biomass are represented by the black points, which are the median estimates, and the vertical lines represent the 95% credible intervals (95% CIs). The pitcher biomass data used to calculate estimated effects are represented by the box plot and colored points behind the marginal effect estimates. (**B**) Predicted effects of functionally distinct community treatments on pitcher biomass. Black points represent median estimates, lines represent the 95% CIs, and color-coded (by treatment) density plots indicate the full posterior. The intercept (dashed posterior) is set as the ACM baseline (estimated biomass = 0.28 g, adjusted to 0). (**C**) Bayesian GLMM marginal effects of treatment on pitcher nitrogen (N) content, and (**D**) predicted effects of bacterial community treatments on pitcher N content, the intercept is set as the ACM baseline (estimated N content = 4.2 mg, adjusted to 0).

To investigate the influence of functionally diverse bacterial communities on plant traits (question i), we evaluated how experimental treatments (CommA, CommB, and CommC) and control treatments (water and acidified cricket media [ACM]) affected pitcher biomass and nitrogen content after the plants hosted the treatments for 8 weeks. We compared our treatment communities to two control groups to help parse out differences between the effect of hosting the bacterial community vs the media they are grown in since pitcher plants may be able to directly absorb amino acids from degraded prey items (35). Pitchers hosting CommB had 1.7 times higher biomass (mean = 0.47 g, 95% CIs = 0.37, 0.60) than pitchers with cricket media only (mean = 0.28 g, 95% CIs = 0.21, 0.36) and 2 times higher biomass than the water-only plants (mean = 0.23 g, 95% CIs = 0.19, 0.30; Fig. 3A and B). We observed that plants that hosted CommB had higher biomass (Fig. 3A and B), longer pitcher length, and higher carbon content (Fig. S4) than not only the water and media controls but also the other experimental treatments, CommA and CommC. While pitchers size varied and the sample size was relatively low due to the slow growth of this perennial plant (*n* = 46), the probability of observing higher pitcher biomass in bacterial community treatments compared to the cricket media (ACM) control was estimated at 81.0% for CommA (standard error (SE) = 0.03), 99.7% for CommB (SE = 0.05), and 84.3% for CommC (SE = 0.04) (based on posterior probabilities from GLM; Fig. 3B). Although the 95% CIs contained zero for CommC, we also saw a trend of a positive effect on total plant growth (measured as the net production of leaves). Plants in the CommC group had an estimated 1.4 greater net (gross new leaves − gross leaf loss) new pitcher production compared to the ACM control over 8 weeks (Fig. S4).

Bacterial communities and the ACM control also affected nitrogen levels in pitcher plants (Fig. 3C and D). Comparing total pitcher nitrogen content between the treatment groups and the two controls (ACM, water), there was a 74% probability that bacterial treatments had higher nitrogen content than the ACM treatment, although the 95% credible intervals included zero. Water had a strong negative effect on pitcher nitrogen content, with plants containing only water having an estimated 2.1 mg of nitrogen (95% CIs = 1.7, 2.6), those with ACM having 4.4 mg (95% CIs = 3.5, 5.6), and those in the three bacterial treatments averaging 4.8 mg (Fig. 3C and D; mean SE = 0.46). We found no impact of the bacterial community treatment groups on other plant traits, including photosynthetic rates and photosynthetic quantum yield (*F*_*v*_/*F*_*m*_) (Fig. S4).

### Compositional differences between the three communities—16S metabarcoding results

**Fig 4:**
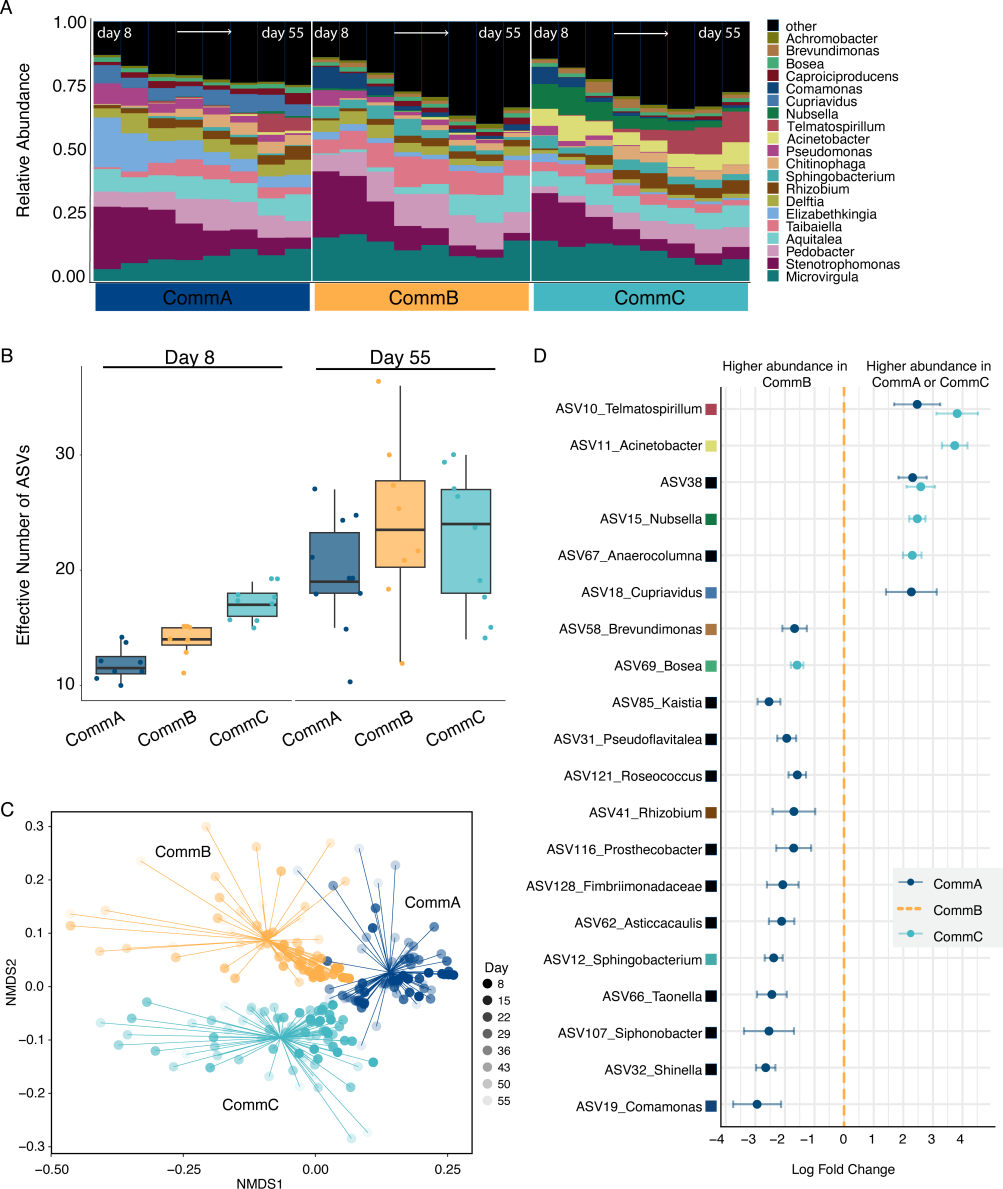
Bacterial community compositional differences between the three bacterial community treatments, based on 16S amplicon sequencing. (**A**) Relative abundance of the 20 most abundant genera from days 8 to 55 (days: 8, 15, 22, 29, 36, 43, 50, and 55), with reads averaged for 10 different pitchers at the same time point. (**B**) The effective number of ASVs (Hill number 1) for each treatment at day 8 and day 55; box plots show the interquartile range, the horizontal lines are the median values, the whiskers extend 1.5 times the interquartile range, and each point represents a pitcher fluid sample. (**C**) Composition-based NMDS was calculated using unweighted UniFrac for all samples within each of the three treatments from days 8 to 55, stress = 0.17, *k* = 2. (**D**) Twenty differentially abundant ASVs, predicted from ANCOM-BC2 (a subset of ASVs with a log fold change greater than ±1.5) with CommB as the reference (orange dashed line). Points are colored by treatment and represent the log fold change of the model effects, with whiskers representing the standard error around each estimate. ASVs are labeled with assigned genus when available, and each colored square matches the color key in panel **A**.

After quality control and rarefaction, we recovered 917 amplicon sequence variants (ASVs) in a community matrix spanning 310 samples (6–10 pitchers per treatment across 8 weekly time points). These ASVs represented 26 bacterial phyla, 192 families, and 286 genera. Among the 20 most abundant genera averaged across pitchers within a treatment at a single time point, *Microvirgula* (14.2%), *Stenotrophomonas* (12.4%), and *Pedobacter* (11.3%) accounted for almost 25% of reads (Fig. 4A).

### Bacterial community diversity and differential abundance

Taxonomic characteristics of the bacterial communities may explain functional differences in our bacterial communities (question i), as we found differences in alpha diversity between the three treatments after 1 week *in planta* (Fig. 4B; Fig. S5). However, the community with the highest richness at week 1 (Comm C) was not the community contributing most to host growth (CommB). Between days 8 and 55, the effective number of ASVs increased (with a mean gain of 7.5 ASVs per sample), and by day 55, the differences in ASV numbers between community treatments had disappeared (Fig. 4B; Fig. S5). Beta diversity was different between the three community treatments (PERMANOVA; *R*^2^ = 0.282, *F*_2, 214_=42.04, *P* = 0.001) using unweighted UniFrac distances, shown as clear clustering of treatments in NMDS visualization and significant pairwise differences over time (Fig. 4C; Table S2). Mantel tests (Spearman’s rank correlation rho) showed significant correlations between the unweighted UniFrac distances of 16S community diversity with chitinase activity (*r* = 0.227, *P* = 0.001) and with final pitcher length (*r* = 0.117, *P* = 0.001), but no significant relationship with protease activity (*r* = 0.004, *P* = 0.44) or pitcher dry biomass (*r* = 0.166, *P* = 0.066).

Analysis of compositions of microbiomes with bias correction (ANCOM-BC) (36) identified 38 differentially abundant taxa between treatments (Table S3) with log fold changes (LFC) greater than 1.5 between at least one pair of treatments (CommB vs CommA, CommC vs CommA, or CommB vs CommC). These taxa were plotted along with error estimates (Fig. 4D). In our model, CommB is set as the reference, so negative log fold change values indicate increased abundance in CommB (Fig. 4D, left side). CommB had a higher abundance of 13 taxa, with 12 of these taxa showing a higher abundance in CommB compared to CommA, and one taxon showing a higher abundance in CommB compared to CommC. Conversely, CommB had a significantly lower abundance in six other taxa (Fig. 4D).

### Linking bacterial physiology with agnostic estimates of functional expression

**Fig 5:**
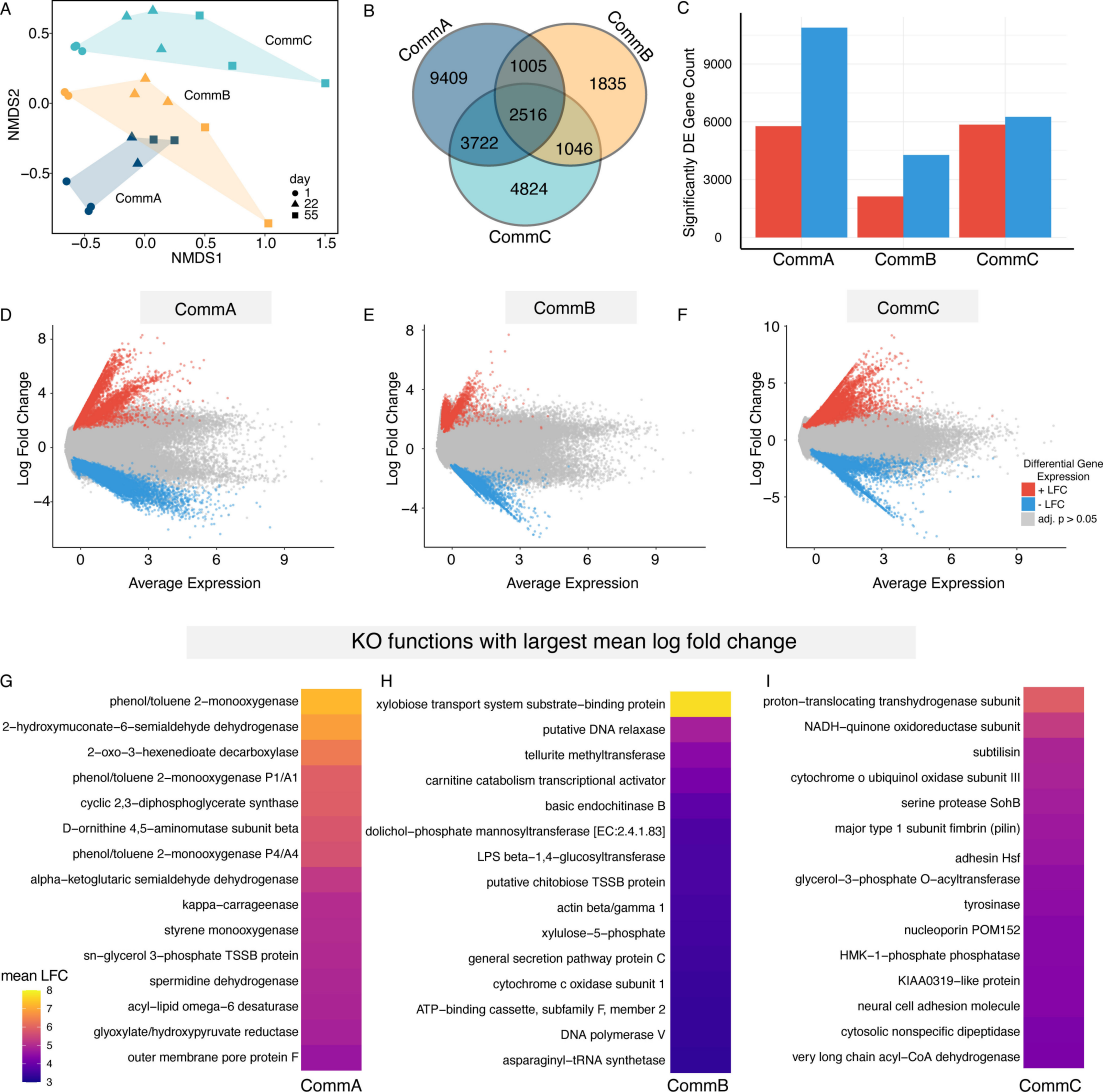
Treatment communities had distinct patterns of gene expression. (**A**) Transcript-based NMDS calculated using Bray-Curtis dissimilarities for all samples within each of the three treatments at days 1, 22, and 55, stress = 0.099, *k* = 2 (PERMANOVA, *R*^2^ = 0.0.20, *F*_2,22_=4.46, *P* = 0.001). (**B**) Venn diagram showing the shared and unique differentially abundant transcripts among the three bacterial communities. (**C**) Bar plot displaying the count of differentially expressed (DE) genes (upregulated in red and downregulated in blue) between the three bacteria community treatments, highlighting significant differences in gene expression between each community based on community contrasts in our differential expression model. The mean abundance (MA) plots depict the log fold change (LFC) versus the predicted average expression of transcripts for each treatment based on our differential expression model, (**D**) CommA, (**E**) CommB, (**F**) and CommC (red points are positive LFC, blue are negative LFC, and gray have an adjusted *P*-value > 0.05). Heat maps illustrating the 20 most abundant KEGG KO functions, based on the differentially expressed genes averaged within each KEGG Orthology (KO) function for each treatment, (**G**) CommA, (**H**) CommB, and (**I**) CommC. The KOs were ranked from high to low mean log fold change.

We identified 218,817 transcripts from 27 metatranscriptomic samples, including 3 pitchers each for 3 bacterial treatments at 3 different time points (Fig. 1). This analysis links bacterial functions directly with differential gene expression (metatranscriptomics), addressing question iii. After filtering out transcripts lacking KEGG orthologs, 162,624 total transcripts remained. We visualized the differences in metatranscriptomic profiles between the three bacterial treatments over time using an NMDS based on Bray-Curtis dissimilarities for all transcripts (Fig. 5A). We found clear differences between treatments (PERMANOVA; *R*^2^ = 0.20 *F*_2, 22_ = 4.46, *P* = 0.001), despite the many shared transcripts, and clear patterns through time (PERMANOVA; *R*^2^ = 0.27, *F*_2, 22_ = 6.00, *P* = 0.001; Fig. 5A). The transcript by sample matrix of ~162,000 genes was normalized and put into our differential expression (DE) model, which identified 24,357 unique significantly differentially expressed genes between our three bacterial communities.

Each treatment had unique and shared transcripts (Fig. 5B). Comparison of differential expression between treatments using pairwise contrasts showed 5,767 transcripts with positive log fold change for CommA and 10,885 with negative log fold change for CommA relative to the mean of CommB and CommC. For CommB, 2,124 transcripts had positive log fold change, and 4,278 had negative log fold change. CommC had 5,851 transcripts with positive log fold change, and 6,257 had negative log fold change, relative to the mean of CommA and CommB. To get an agnostic view of functional expression between treatments, we plotted the model estimate of mean average expression and log fold change for each transcript in each treatment contrast, and color-coded according to *P*-value (Fig. 5D to F). We averaged the log fold change for transcripts mapping to the same KEGG function and ranked them from high to low to find the most differentially expressed functions in each treatment (Fig. 5G to I).

**Fig 6:**
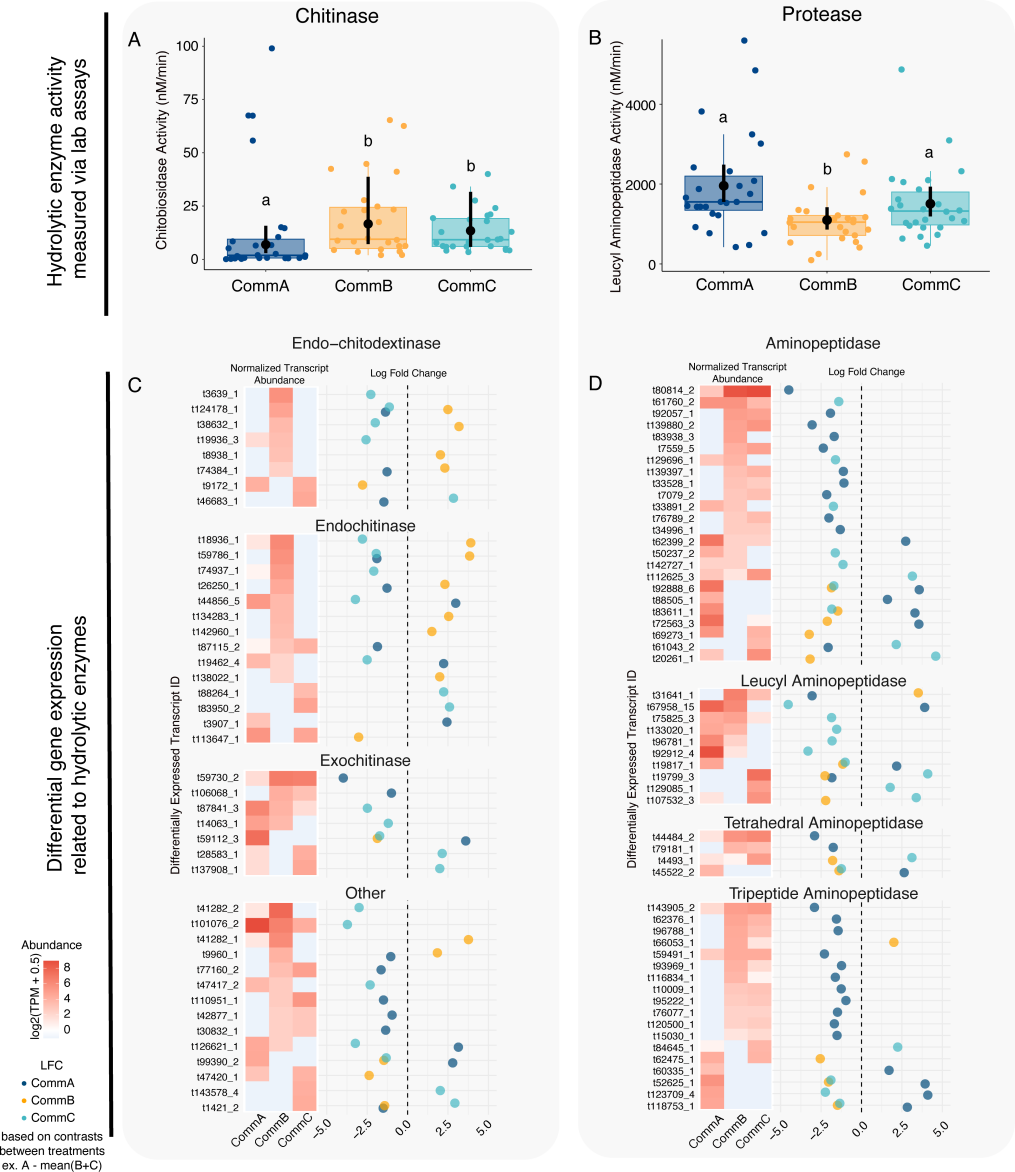
Functional assays of hydrolytic enzyme activity generally matched well with transcript levels of genes related to those functions. (**A, B**) Enzyme activity was measured in communities from nine pitcher plants whose samples also underwent downstream metatranscriptomic analysis (within a treatment, three plants × 9 weeks, *n* = 27). Box plots and colored points represent the raw data, with the horizontal bar representing the median values and whiskers showing 1.5 times the interquartile range. The black points represent the median marginal effects from the model testing the effect of treatment enzyme activity with week as a random intercept, the black vertical bars represent the 95% credibility intervals around each estimate. Chitinase- and protease-related transcript comparisons: within each panel, log fold change (LFC; right) and normalized transcript abundance (TPM; left) of transcripts associated with chitin metabolism KOs (**C**) and protein metabolism KOs (**D**) determined from significantly differential transcript abundances. The log fold change points are colored by treatment and represent the effect size from the differential expression model. The absence of a point for treatment indicates that the transcript is not differentially expressed for that contrast.

In CommA, we found high expression of functions related to phenol/toluene 2-monooxygenase, aminomuconate-semi aldehyde, and 2-oxo-3-hexenedioate decarboxylase, related to aromatic compound degradation (Fig. 5G). For CommB, we found high expression of functions for xylobiose transport system substrate-binding protein, basic endochitinase B, and fructose-6-phosphate phosphoketolase (Fig. 5H). We found support for our physiological measures of high chitinase activity (Fig. 1A and 6A), with basic endochitinase B being one of the top five highly expressed functions for CommB. For CommC, there was high expression of functions related to proton-translocating transhydrogenase, glycerol-3-phosphate O-acyl transferase, and very long chain acyl-CoA dehydrogenase, related to fatty acid metabolism (Fig. 5I).

### Linking bacterial physiology with hydrolytic enzyme functional expression

Beyond the general functional differences identified by differential transcription, specific functions related to hydrolysis were examined. We measured chitinase as chitobiosidase and protease as L-amino-peptidase enzyme activities, analyzing only the nine pitchers that also had metatranscriptomics (3 of 10 plants per treatment). Over 8 weeks, CommA had lower chitinase activity, compared to CommB and CommC (Fig. 6A; Fig. S6). This result was similar to those of the physiological assays in the bacterial communities prior to inoculation (Fig. 1A), but in this smaller subset across time, CommB and CommC were not statistically different. Protease activity in the nine pitchers was consistent with community assays prior to inoculation, with CommB showing lower protease activity than CommA and C (Fig. 6B; Fig. S6).

We used metatranscriptomic data to explore hydrolysis functions in two ways. First, we investigated the transcript abundances for differentially expressed genes that mapped to chitinase or protease functional KOs. Second, we paired these abundance measures with the log fold change predicted by our DE model. Within this model, contrasts were created to compare the expression levels of each community treatment against the average of the other two (see Fig. 6 legend and Materials and Methods). This allowed us to identify genes that were differentially expressed in one treatment relative to the others. In Fig. 6C, we show all significant DE genes mapping to chitin degradation or transport KOs, including endochitodextinases, endochitinases, exochitinases, and chitin transport systems and putative chitinases. CommB had higher transcript abundances of these chitin-related genes, including 12 genes with higher expression in CommB, compared to only six chitin-related genes with higher expression in CommA, and only seven in CommC. Similar patterns emerged with normalized transcript abundance for these same genes (log-transformed transcripts per million) (Fig. 6C, left side). In Fig. 6D, we show many significant DE genes mapping to protein degradation, including general aminopeptidases, leucyl aminopeptidases, tetrahedral aminopeptidases, and tripeptide aminipeptidases. CommB had lower expression of genes related to protease enzymes, two genes with higher expression in CommB, compared to 12 protease-related genes with higher expression in CommA, and seven genes for CommC.

### Linking bacterial physiology with carbohydrate and amino acid metabolic functions

Carbon substrate use profiles were significantly different among all pairwise combinations of the three treatments on both day one and day 55 (PERMANOVA pairwise differences, *P* < 0.05; Fig. S3; Table S1). Out of the 31 carbon substrates tested, the three communities exhibited differential capacity to use nine (Fig. S7 and S8). CommB had statistically higher use of N-acetyl glucosamine, beta methyl glucoside, cellobiose, and pyruvic acid methyl ester. CommA showed the highest use of Tween 80, serine, and threonine. Meanwhile, CommC exhibited higher use of galactonic acid, gamma lactone, and glucosaminic acid.

Relating carbon substrate use from EcoPlate functional assays, we identified significantly differentially abundant transcripts related to carbohydrate and amino acid metabolism (Fig. S7B and C) and aggregated transcript counts by KO function. Because CommB had the largest positive effect on pitcher biomass, we highlighted functions where CommB exhibited markedly higher expression than either CommA or CommC by at least log_2_ = 4 transcripts per million (TPM). We identified 54 KOs that were higher in CommB, many of which are related to the metabolism or transport of carbohydrates like fructose, glucose, and chitin. In addition, we identified 33 KOs that were higher in CommB, including the enzymes urea carboxylase, nitrile hydratase, and argininosuccinate lyase. These are related to N cycling, contributing to the breakdown and recycling of nitrogen-containing compounds (37, 38).

### Connecting community functions to metagenome-assembled genomes

**Fig 7:**
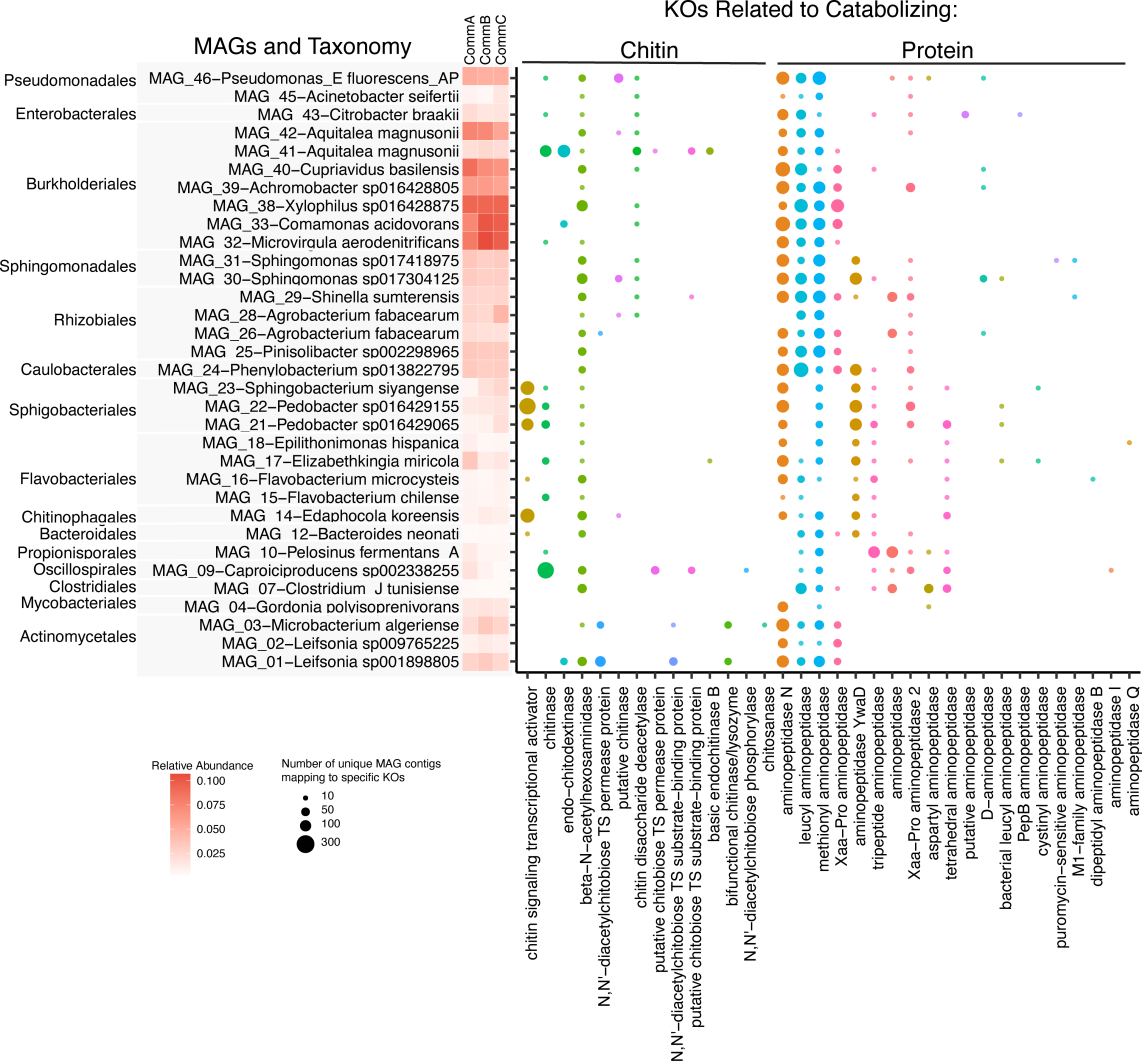
MAGs enriched in functions related to chitin and protein metabolism. The size of the bubble represents the number of unique MAG contigs mapped to functional transcripts (RNA sequences from the same samples) grouped by KEGG orthology (KO; *x*-axis) and filtered to include only those KOs associated with targeted hydrolytic enzyme functions (chitin and aminopeptidase metabolizing KOs). The *y*-axis is ranked by phylogenetic relatedness and shows the MAG identifier along with the associated taxonomy and a heatmap of the relative abundance of each MAG in the three community treatments.

In addition to the 16S amplicon sequencing to identify taxa present, we conducted shotgun metagenomic sequencing of 18 samples (three plants per treatment at day 1 and day 55). The resulting data were used to quantify taxonomy and build high-quality metagenome-assembled genomes (MAGs), which were then connected to the metatranscriptomics data, focusing on hydrolytic enzyme functions. Taxonomic identifications of MAGs were similar to the 16S amplicon results, with some differences likely due to different databases queried for the two data types (Fig. S8). We built 36 MAGs, of which 26 were high quality, 8 were medium quality, and 2 were discarded due to redundancy >10% (Table S4). We calculated the relative abundance of each MAG in each treatment community, mapped the functional transcripts to the MAGs, and selected the KOs associated with chitinase or aminopeptidase (proteinase) activity (Fig. 7). In Fig. 7, each bubble represents the number of unique MAG contigs that mapped to each KO, identifying 13 chitin metabolism KOs and 20 aminopeptidase-related KOs. MAG_09 (*Caproiciproducens* sp.) had the highest number of functional chitinase-associated KOs, followed by MAG_41 (*Aquitalea* sp.), and MAG_21, MAG_22, and MAG_23 (order Sphingobacteriales). Protease activity was well represented across the MAGs, with the highest abundance of aminopeptidase-related KOs associated with MAG_24 (*Phenylobacterium* sp.), followed by MAG_29 (*Shinella sumatrensis*), MAG_33 (*Comamonas acidovorans*), and MAG_30 (*Sphingomonas* sp.).

## DISCUSSION

### Bacterial community functioning influences plant traits

Plants and microbes have been associated for approximately 450 million years (39). Over this vast period, many microbes have evolved beneficial interactions with their hosts; however, our understanding of the mechanisms behind these interactions remains limited. In this study, bacterial community function significantly influenced host growth and nutrient acquisition in the pitchers of carnivorous pitcher plants. The biomass of pitchers hosting a specific microbial community almost doubled relative to controls, and we have identified potential functional drivers of this difference. We also found positive effects of CommB on pitcher length and carbon content. Together, these results addressed our initial question of whether functionally different bacterial communities have varying effects on host plant traits. Functional differences among the bacterial community treatments, such as chitinase activity, were correlated with pitcher growth and plant nutrient acquisition. Many studies provide evidence for how the metabolites produced by microbes incite benefits to their host plants, for example, plant growth-promoting hormones (19, 40, 41). However, to the best of our knowledge, this is the first example connecting collective microbial community function to host nutrient content and growth using metabarcoding, metagenomics, and metatranscriptomics.

This research leveraged a unique model system, with microbes hosted in aquatic microecosystems inside pitcher-shaped leaves, in contrast to classic non-aquatic phyllosphere systems. One might expect that the aquatic pools would have a lower impact on host health than more intimate associations such as root and leaf-associated microbial communities. However, the positive effect of bacterial communities on leaf biomass *in planta* in just over 8 weeks demonstrates a rapid response to their microbial communities for these slow-growing, long-lived plants (Fig. 3A). In this experiment, a single pitcher was responsible for nitrogen acquisition for the whole plant, the other pitchers in the experimental plants were filled with sterile water so could not access nitrogen. Nitrogen was likely translocated out of the target pitcher to support other pitchers (29, 42), reducing the sensitivity or size of the effect on just the measured target pitchers. These plants generally have low nitrogen content compared to other plants (43), suggesting that even small increases in nitrogen acquisition could significantly improve their growth and fitness (Fig. 3) (40). In addition, several plant traits were examined, and we found that the percent of carbon and nitrogen in the target pitcher was highly correlated with the second-newest pitcher (Fig. S10), which was not inoculated or provided with ACM, indicating that nutrient translocation was likely occurring. Our findings demonstrate that functionally different microbial communities can influence host plant health, most likely via the release and supply of nutrients that enhance plant growth and fitness.

### Relationship between bacterial composition and function

Recent studies have linked microbially mediated plant benefits to specific taxa. For example, in an experiment with synthetic bacterial communities, pathogen suppression in *Arabidopsis* was driven by three specific bacterial taxa (44). By contrast, other studies have observed a community-level effect, such as the positive influence of microbial communities, but not individual strains, on duckweed growth (45). Our study supports a combined community effect, as we did not see the presence or absence of specific taxa driving main effects (Fig. 4). One possible explanation could be the importance of microbe-microbe interactions, metabolic collaboration in microbial communities is common, making the effects of single taxa less likely (39, 45, 46). Addressing our second question, although we found clear taxonomic and functional differences in our bacterial communities, most ASVs and MAGs were shared across all three communities and the differences were in rare taxa (Fig. 4 and 7), without specific taxa appearing to drive primary functional differences. Observed differences in hydrolytic enzyme activity and substrate use were likely affected by small differences in relative abundances of functionally important taxa across our communities or differences in gene expression, indicating that community functions are not solely determined by composition but, instead, specific combinations of taxa leading to distinct functional profiles (Fig. 6). Corroborating our findings, a recent study with wild pitcher plants found significant functional differences in the microbial community with modest microbial compositional changes following an experimental manipulation, suggesting that differential gene expression, not composition, drove functional change (47). Similarly, in lake microbial communities, functional differences were only weakly related to taxonomic composition (48). Plants often host high bacterial biodiversity, providing complex habitats and resources that create bacterial niches (49). In pitcher plants, the colonization of pitchers by microbial communities is influenced by factors like season, host characteristics, and nutrient inputs, and these differences in microbial community composition likely have important implications for community function (35, 50, 51). In this study, we demonstrated that relatively small differences in bacterial composition and abundance within pitchers can have significant consequences for nutrient cycling from captured prey supplied to the host plant.

### Coupling measured bacterial functions with differential gene expression

Our agnostic examination of total gene expression allowed us to first examine general functional differences across the three bacterial communities. While substantial differences in hydrolytic enzyme activity were observed between bacterial communities after some time *in planta*, these did not fully explain the observed benefits to plant biomass and pitcher nutrient acquisition, suggesting a more synergistic, collective community effect rather than the additive effect of transcription of selected genes. Each community exhibited differential transcription of a large number of unique genes (Fig. 5), many of which are involved in producing enzymes involved in breaking down complex substrates. Interestingly, despite this untargeted approach, CommB had two chitinase-related KOs among its highest transcribed functions, further supporting this as an important function for liberating nutrients including nitrogen from chitin (52). In addition, many of the most highly transcribed genes found in CommB mapped to KOs relating to breaking down xylobiose, carnitine, and chitin, indicating this community is efficient at metabolizing complex organic nutrients with probable benefits to the host plant and other members of the microbial community. The catabolism products of these substrates have been found to trigger plant responses, including defense and growth (53, 54). Lastly, while not in the top 20 KO functions, the ACC deaminase gene was significantly more expressed in CommB. This enzyme plays an influential role in reducing ethylene-mediated stress, which can have cascading benefits on growth and development (40). Together, these findings suggest that differential gene expression between microbial communities, due to subtle differences in composition, can contribute to measurable differences in host plant growth.

### Targeted bacterial functions influence plant traits

Few studies have successfully linked direct enzyme activity with transcriptomics for whole microbial communities. Bacterial communities play pivotal roles in nutrient transformations, so to better understand community functional dynamics, we linked bacterial physiology with hydrolytic enzyme expression to investigate how chitinase and protease activities *in planta* related to differential gene expression for these functions. CommB and C exhibited higher chitinase activity in physiological assays, and CommB also showed significantly higher expression of genes related to chitin degradation in our metatranscriptomic analyses, connecting active enzyme activity to gene expression (Fig. 6). By contrast, CommB had lower leucyl aminopeptidase activity measured by laboratory assay, while differential gene expression identified many genes mapping to aminopeptidase enzymes expressed in CommB. These results provide evidence that while culture-based physiological assays are valuable, they fail to completely characterize total nutrient cycling activities present in a community. Pairing physiological assays with metatranscriptomics built a clearer picture of the functional activities present in our communities.

The analysis of carbohydrate and amino acid metabolism across the three bacterial communities supports our findings on hydrolytic enzyme activities, revealing distinct organic substrate preferences among the communities (Fig. S7). CommB exhibited higher utilization for substrates like N-acetyl glucosamine (GlcNAc) and cellobiose. GlcNAc is a monomer of chitin, chitobiose, or chitotriose and is used for many critical metabolic functions within bacterial cells (55–57), while cellobiose is a metabolic precursor to glucose and interacts with β-glucosidase to yield two glucose molecules used in glycolysis (58). Increased transcription of genes related to metabolizing these substrates provides further evidence that CommB is efficiently cycling diverse nutrients in this community, products which are likely important for not only community cross-feeding but for nutrient supply to the host plant. Although it has long been assumed that bacterial community enzyme activity is a key mechanism in pitcher plant nutrient acquisition (25, 29), our results are the first to directly measure and provide evidence for this mechanism.

### Conclusion

In this controlled study, plant growth was enhanced by the collective function of a specific bacterial community. The most growth was observed in pitchers hosting a bacterial community with high chitinase activity and high differential expression of genes related to nutrient cycling, supporting the release of nutrients for uptake by the host plant. We characterized collective community composition and function using a suite of complementary approaches that highlighted the complexity and dynamics of plant-microbe interactions and the role microbial communities can play in host health and growth. The benefits of plant-microbe interactions to host productivity are likely often not driven by a single functional parameter, nor a single taxon expressing a gene encoding a specifically useful function. This bacterial community analysis suggests that measurable functional traits can identify relevant bacterial effects in phyllosphere communities. Identification of these relationships contributes to increased knowledge of the effects of bacterial communities on host plant traits and can inform other systems with applications in agriculture, conservation, and restoration.

## MATERIALS AND METHODS

See supplemental methods for greater detail.

### Experimental design

Mature *Sarracenia purpurea* greenhouse plants (donated by T. Miller, FSU) were cleaned and repotted into autoclaved media (7 parts milled sphagnum peat:3 parts sand) and bleach-sanitized pots. Plant surface decontamination consisted of washing the leaves and roots in distilled water prior to soaking in 1% hydrogen peroxide for 15 minutes, this process was repeated three times. Plants were transferred into clean plant growth chambers (Hoffman Mfg. Inc., Corvallis, OR, USA; SG2-22) and allowed to acclimatize for 12 weeks prior to the start of the experiment. Care was taken to keep the plants, pots, and all contact with the growth chambers as sterile as possible; however, chambers were supplied with ambient unfiltered air through their vents and when the chamber doors were open.

The three distinct bacterial communities (CommA, CommB, and CommC) used for inoculation were previously collected from wild *S. purpurea* pitcher plants (35). These communities were selected from the 10 microcosm communities used by Bittleston et al. (35) (CommA = M01, CommB = M06, CommC = M09). All 10 communities from that study were reconstituted from cryogenic culture and grown in the laboratory using acidified cricket media (ACM) with 1:1 serial transfer into fresh media every 72 hours for several months. Cricket media was used to establish the bacterial communities and was prepared by acidifying (pH = 5.6, 1.0 M HCl) ground food-grade house cricket media (3 g/L H_2_O; Thailand Unique Inc.) and then autoclaving. The three communities (CommA, CommB, and CommC) were selected based on their differences in chitinase and protease activity (Fig. 2A and B). In the previous study using these communities, CommA (M01) was dominated by an *Aquitalea* species. In our study, this community was dominated by an *Elizabethkingia* species, but an *Aquitalea* species became the second-most abundant species by the end of the experiment. CommB (M06) was dominated by a *Comamonas* species, which is consistent with previous work. Lastly, CommC (M09) also had a high abundance of a *Comamonas* species which diverges from the previous study, possibly due to shifts in community composition due to freeze-thaw dynamics. The bacterial taxa in these samples are representative of natural pitcher plant microbiomes, while lower in diversity, they represent some of the most common and abundant taxa found in environmental samples (27, 47). In addition to three experimental bacterial communities, two experimental controls were ACM only and sterile deionized water only. Both controls contained no bacterial cultures, the ACM control was used to test the effects of nutrient media without any bacteria on plants. The water control examined the effect of sterile ACM media with no bacteria.

Ten individual pitcher plants were randomly assigned to each bacterial treatment group and six to each control group, for a total of 42 individual plants. In general, the selected pitchers were roughly the same size, and no statistical differences (95% CIs cross 0) in pitcher length were observed on day 1 between plants in the five treatments (mean = 7.04 cm _ACM_, 8.07 cm _CommA_, 5.81 cm _CommB_, 7.12 cm _CommC_, 5.79 cm _Water)_. On day 0, on each plant, one new, unopened pitcher was manually opened using sterile gloved hands and carefully inoculated with a bacterial community culture or control treatments until each pitcher was 3/4ths full. For example, if a pitcher held 8 mL when 3/4 full, it received 8 mL sterile ACM and 0.8 mL bacterial community culture. Due to varying pitcher sizes, some smaller pitchers received lower volumes but in the same medium-to-culture volume ratio. The single pitcher on each plant that received one of the five treatments was the target pitcher (pitcher 1). All other pitchers on each plant received sterile water for the duration of the experiment. For some plant trait measurements, data were also collected on the second-newest pitcher (pitcher 2). We found no statistical differences in bacterial cell concentration (from CFU counts) between our three cultures prior to inoculation (mean = 2.18 × 10^9^ colonies/mL, *F*_2,3_=5.857, *P* = 0.09), so pitchers were inoculated with similar bacterial densities.

### Sample collection

Samples were collected weekly, starting the day after inoculation (day 1) 4.0 mL was collected from each leaf using a sterile technique. This sample was then aliquoted, 1.5 mL was frozen in sterile screw cap tubes for amplicon sequencing, 750 µL was frozen in cryotubes in liquid nitrogen for metagenomic/metatranscriptomic analysis, 750 µL was mixed with 750 µL 80% sterile glycerol and frozen in screw cap tubes for culture preservation. All preserved samples were stored at −80°C until nucleic acid extraction. The remaining 1.0 mL was used in the microbial physiological assays described below. The full 4.0 mL was not removed from very small pitchers on day 7 due to limited volumes, but in all cases, pitcher size increased very quickly in the first week and full samples were collected at future samplings. In all plants, the sample volume (4.0 mL) was replaced with sterile cricket media as a weekly food source, and an additional 2 mL sterile water was added to pitcher 1 to replace evaporated fluid (total added liquid each week to all plants = 6.0 mL). There was some damage that occurred to some pitchers during the course of the experiment, plants 25 (CommB), 44 (CommB), 51 (CommC), 9 (CommC), and 29 (CommA) were removed from all analyses.

### Bacterial physiological assays

Chitinase and protease activities were quantified weekly on a sample from each treatment pitcher using fluorometric assays in black 96-well microplates (30, 59, 60). Fluorescence emission was measured every 5 minutes for 1 hour using a microplate reader (Biotek Synergy Mx Multi-Mode Microplate Reader). Of three types of chitinases found in the *S. purpurea* pitchers communities, chitobiosidase activity was selected for quantification because preliminary data showed higher activity potential compared to β-N-acetylglucosaminidase and triacetyl chitotriosidase. Chitobiosidase (hereafter chitinase) activity was measured using 200 µL of sample (culture or pitcher fluid) and L-amino peptidase (protease) activity was measured using 50 µL of sample according to previously published methods (61). The “functional fingerprint” for each bacterial community was measured by examining the community’s capacity to use 31 carbon substrates. These measurements were conducted on the first and last day of the experiment (days 1 and 55) using Biolog EcoPlates, full methods also follow those previously published (61). After an initial analysis, extreme outliers at day 55 were removed from the EcoPlate analysis, including plant_id 34 and 15 from CommA, 27 from CommB, and 28, 21, and 41 from CommC.

### Plant functional traits

Pitcher morphological traits (62), including length, width, and opening aperture (60), were measured weekly for 8 weeks (Fig. 1). For each plant, the number of mature leaves was recorded at the beginning and end of the experiment. The length of each pitcher (rhizome to tip) was measured with a flexible tape (cm) and the pitcher diameter (at widest point), and aperture of the pitcher, will be measured with digital calipers (mm). Tools were carefully sanitized with 70% ethanol between each measurement. At the end of 8 weeks, two pitchers were cut at the base for biomass measurements, the target pitcher (pitcher one) and a secondary mature pitcher (second-oldest pitcher, pitcher two). Biomass was recorded before and after drying the plant tissue at 65°C for 72 hours. Plant tissue was homogenized in an electric spice grinder and powdered samples were analyzed using a CN elemental analysis (Flash EA1112), the proportion of carbon and nitrogen for each tissue sample was calculated using the mass of the nutrient divided by the mass of the ground sample.

### 16S rRNA sequencing and analysis

To characterize bacterial community composition, DNA was extracted from all samples (CommA, CommB, CommC, ACM & water controls, on days 8, 15, 22, 29, 36, 43, 50, and 55, *n* = 310) using the DNAdvance Genomic DNA Isolation Kit (Beckman Coulter A48705). Samples (750 µL) were bead beaten in lysis buffer at 2,400 RPM for 10 min and then incubated at 55°C shaking (150 RPM) overnight before proceeding with the extraction (halving all the reagents but following the protocol per the manufacturer’s directions); each 96-well plate included one negative control. DNA was quantified using the AccuClear Ultra HS dsDNA kit (Biotium #31028). 16S rRNA gene amplification and library preparation and sequencing were conducted by the Environmental Sample Preparation and Sequencing Facility at Argonne National Laboratory on a 151 bp × 12 bp × 151 bp Illumina MiSeq run targeting the V4 region of 16S rRNA using the 515F and 806R primers (63).

The 16S rRNA gene sequences were processed in QIIME2 (version 2022.8) (64), demultiplexed using no-golay error correction, and quality filtered to remove reads with a mean score less than 20 and trimmed to the sequence length of 150 base pairs. The DADA2 module was used to denoise sequences and generate ASVs (64, 65). Taxonomy was assigned using the classify-sklearn method which is a native Bayes classifier, and a pre-trained classifier made with Silva v. 138 database containing 99% ASVs from 515F/806R region (66). The phylogenetic tree was built using multiple-sequence alignment via SEPTT and phylogenetic reconstruction via FastTree, both via a QIIME2 plugin (67, 68). Taxonomy and ASV tables were migrated to R for further analysis and visualization using qiime2R, picante, and phyloseq (69–71).

Across 316 samples (310 pitcher fluid samples and 5 negative controls) DADA2 generated 2,633 ASVs. Contaminant ASVs were identified and discarded using the decontam R package (72), the decontam method “prevalence” was used which identified ASVs based on their presence and abundance in our negative controls. Data were quality filtered to remove non-prokaryotic ASVs and ASVs classified as mitochondria or chloroplasts, and only include observations with at least 10 sequences and samples with at least 1,000 sequences, resulting in 310 pitcher fluid samples (day 8 through day 55) with a cumulative 1,036 distinct ASVs, with 25,317 mean reads per sample (min reads/sample = 1,669; max reads/sample = 57,927). Samples were rarified down to 1,669 reads, resulting in 310 samples with 1,036 ASVs. A negative binomial model was built using the brms package to investigate the effect of a treatment group on alpha diversity (73).

For ASV-level differential abundance explained by the treatment groups, an analysis of compositions of microbiomes with bias correction (ANCOMBC2) was used, this is a Bayesian statistical approach that accounts for sampling fraction, normalizes the read counts, and controls for false discovery rates (fdr) (36). Quality controlled but unrarefied ASV counts were analyzed using the ANCOMBC function, the adjusted *P*-value method was set to fdr, prevalence (prv_cut) was set to 0.3, the level of significance (alpha) was set to 0.05, pairwise directional test (pairwise) was set to TRUE, with the rest of the parameters left as default. Significantly differentially abundant ASVs were identified across the treatment groups and through time.

### Metagenomic and metatranscriptomic sequencing and analysis

The pitcher fluid from 3 of the 10 pitchers in each treatment group was selected from three time points (3 plants × 3 treatments × 3 timepoints [days 1, 22, and 55], *n* = 27) for simultaneous DNA/RNA extractions for metagenomic and metatranscriptomic sequencing using the Qiagen AllPrep Bacteria DNA/RNA/Protein Kit (Cat 47054), according to the manufacturer’s directions. Briefly, flash-frozen samples were thawed and pelleted, pellets were resuspended in lysis solution and bead beat for 10 minutes at 2,400 RPM after which 350 µL of lysate was transferred to a spin column where the DNA was collected in the column and the flow through was further processed for RNA extraction. Samples were eluted in nuclease-free water and stored at −20°C until quantification and library preparation. Nucleic acids were quantified using a fluorometer (Qubit, Invitrogen). Libraries were prepared for the full 27 RNA samples using NEBNext rRNA Depletion Kit (NEB#E7850) and NEBNext Ultra II Directional RNA library prep kit for Illumina (NEB#E7760) using the manufacturer’s protocols. Metagenomic libraries (DNA) were prepared for a subset of these samples (3 plants × 3 treatments × 2 timepoints [days 1 and 55], *n* = 18) using NEBNext Ultra II FS DNA library prep kit for Illumina (NEB#78055). Sequencing was done on a NovaSeq partial lane to a sequencing depth of 55 Gb per library at Novogene Co. (Sacramento, CA, USA).

Shotgun metagenomic and metatranscriptomic sequences were processed on BSU’s Borah cluster. Adapters and low-quality sequences were trimmed using Trimmomatic with the following settings: SLIDINGWINDOW:4:20 MINLEN:25 ILLUMINACLIP:TruSeq3-PE.fa:2:40:15 based on the FastQC (v0.12.1) and viewed with MultiQC (v1.6) quality reports (74–76). Metatranscriptomic sequences were further processed to remove ribosomal RNA using the default settings (-t 4, -l 150, -e rrna) in Ribodetector (77). Two co-assemblies were produced (metagenomic and metatranscriptomic) using MEGAHIT (v1.2.9) (78).

For the metagenomic analysis, sourmash was used to assign and quantify taxonomy using the full GTDB database (79, 80). Contigs in the coassembly and paired-end reads were binned using MAXBIN2, CONCOCT, and Metabat2, consensus bins were produced using DASTool (81–84). Completeness and contamination of each MAG were calculated using the presence of single-copy marker genes with CHECKM2 (85). Comparisons and dereplication of MAGs employed dREP (86). Taxonomy was assigned to the quality-controlled MAGs using the full GTDB database within sourmash. Bins were quality ranked according to their completeness (87, 88). The abundance of each MAG in each treatment was calculated by first building an index for each MAG using bwa and samtools to align the individual forward and reverse sample reads to each MAG (89, 90). The phylogenetic tree for the MAGs was built by first extracting the marker genes from each MAG using CHECKM2 and then multiple sequence alignment with MAFFT (85, 91). The phylogenetic tree was built with IQ-Tree (92) and parsed in R. Predicted gene sequences from the metatranscriptomic samples were mapped to a custom database built with the MAGs, and then used blastn (93) to quantify the number of contigs in each MAG that matched with each predicted gene.

For metatranscriptomic analyses, we used SeqKit and VSEARCH to look at the quality of coassembly before and after chimera removal (94, 95). We removed 23 contigs that surpassed the 50,000 base pair threshold. Open reading frames (ORFs) were predicted using Prodigal (v2.6.3) (96). We used CDHIT to remove redundancy in our predicted proteins by clustering transcripts (97). Transcript abundance quantification was done in Kallisto (v1.9.0) and resulted in transcripts per million (TPM) and counts for each transcript and sample (98). KOFAMSCAN (1.3.0) was used to bin ORFs into KOs using the full KEGG database (99).

### Statistical analyses

All statistical analyses were conducted in R version 4.2.2 (100). Alpha diversity metrics (Hill Number 1) were calculated using the vegan package and additional processing used tidyverse and janitor (101–103). Community similarity in ASVs and EcoPlate carbon substrate use among samples in each treatment group was determined using the vegan package (101). Compositional differences (beta diversity) and changes across the three bacterial community treatments from days 8 to 55 were assessed using phylogenetic methods, unweighted UniFrac (104). Mantel tests assessed correlations between bacterial composition and hydrolytic enzyme activity or pitcher biomass (105). To visualize the similarity in EcoPlate carbon substrate use among samples and treatments, a metaMDS analysis with Bray-Curtis distance at two dimensions (*k* = 2) was utilized. In both cases, non-metric multidimensional scaling (NMDS) visualized the dissimilarities calculated between each pitcher fluid sample. A non-parametric multivariate analysis of variance test (PERMANOVA, adonis2 function, by=“margin”) was used to assess any differences between treatments, and pairwise adonis was used to assess the effects of treatment and time while accounting for repeated measures for each pitcher (101). Dispersion, or the homogeneity in dispersion between treatments, was calculated using the betadisper function (permutations compared using a *post hoc* Tukey test (101).

Metatranscriptomic statistical analyses began with a matrix of filtered genes, samples were normalized using the calcNormFactors and differential transcript abundance was quantified using Dream within the variancePartition package (1.28.9) (106). To link specific taxa to differentially expressed functions, significantly differentially abundant contigs were mapped to the MAGs using a custom database built from the MAG contigs and BLAST (2.14.0) (93).

Bayesian hierarchical models using a gamma (log-link) GLM were fitted using the brms package and visualized using bayesplot, ggeffects, and tidybayes (73, 107–110). Bayesian GLMs are robust to small sample sizes and outliers, and the gamma distribution best fits the data-generating processes (111). The continuous predictor variables were standardized and mean centered using the standardize package (112). In all models that contained the treatment group as a predictor, we set ACM as the baseline, to compare the effects to pitchers with food but no inoculated bacteria. Default uninformative priors were used, convergence and mixing of chains and unimodality in posterior predictions were visually assessed, and all R-hat values were close to 1.0. The model fit was evaluated using the posterior predictive check function in brms. Full model specifications for all analyses are found in our public repository: https://github.com/jessibernardin/microbial-function-plant-trait.

## Data Availability

Scripts and data associated with this paper are available at https://github.com/jessibernardin/microbial-function-plant-trait. The raw reads for 16S rRNA amplicon sequencing, raw reads for whole-genome shotgun sequencing, whole-genome metatranscriptomic sequencing, and nucleotide sequences of MAGs have been deposited in the National Center for Biotechnology Information (NCBI) under the project number PRJNA1028624.

## SUPPLEMENTAL MATERIAL

Supplemental Material msystems.01298-24-s0001.docx Supplemental methods, figures, and tables.

ASM does not own the copyrights to Supplemental Material that may be linked to, or accessed through, an article. The authors have granted ASM a non-exclusive, world-wide license to publish the Supplemental Material files. Please contact the corresponding author directly for reuse.
