# Supplementary material for: Bacterial community function increases leaf growth in a pitcher plant experimental system
Source: mSystems. 2024 Nov 25;9(12):e01298-24. doi: 10.1128/msystems.01298-24 (PMC11651108; doi:10.1128/msystems.01298-24)
Supplement: Supplemental Material — Supplemental methods, figures, and tables. [file msystems.01298-24-s0001.docx]

Supplementary Information for:

Bacterial community function increases leaf growth in a pitcher plant experimental system

Jessica R. Bernardin, Erica B. Young, Sarah M. Gray, Leonora S. Bittleston

Corresponding author: Jessica R. Bernardin: jessicabernardin@boisestate.edu

This PDF file includes:

[**Supplementary Methods** 2](#_Toc178074982)

[Growth Chamber Conditions 2](#_Toc178074983)

[Experimental and Plant Growth Details 2](#_Toc178074984)

[Bacterial Community Respiration Methods 2](#_Toc178074985)

[Plant Photosynthesis Methods 3](#_Toc178074986)

[Plant Traits Used 3](#_Toc178074987)

[ANCOM-BC 3](#_Toc178074988)

[Model Structure Detail 4](#_Toc178074989)

[Metagenomic Analyses Details 4](#_Toc178074990)

[Metatranscriptomic Analyses Details 4](#_Toc178074991)

[**Supplementary Figures** 6](#_Toc178074992)

[**Supplementary Tables** 18](#_Toc178074993)

Other Supplementary Materials for this manuscript include the following:

Github: https://github.com/jessibernardin/microbial-function-plant-trait

Zenodo: <https://doi.org/10.5281/zenodo.13145643>

NCBI: https://www.ncbi.nlm.nih.gov/bioproject/PRJNA1028624

# **Supplementary Methods**

## Growth Chamber Conditions

The chambers were configured with a diel light and temperature cycle, 14-hour photoperiod (Photosynthetic photon flux density (PPFD)= 62 μmol m^-2^ s^-1^) at 31 °C and 10 hours dark at 17 °C, with relative humidity at 70%. Additional temperature probes were added to each chamber to monitor chamber conditions.

## Experimental and Plant Growth Details

Because plants did not all have new, unopened pitchers in the same week, new plants were added to the experiment in stages, once a week for 5 weeks until all plants had been inoculated. To ensure there was no effect of this staggered addition of plants, plants were assigned to treatment groups randomly and sample analysis was based on time after inoculation, not date. The cultures for the three bacterial communities were maintained (as noted above) in the lab and added each week to the appropriate plants. There was no change in bacterial community composition in these cultures, and we found that the community composition of samples within the same treatment, but added on different weeks, had very tight clustering 7 days after inoculation, regardless of the date added. Every 2 days, distilled water was added to the trays under the pots, to prevent splashing and contamination into pitchers. Each week, the plants were rearranged within the chamber and rotated according to a randomization map created using a random number generator.

**Bacterial Community Growth Rate Methods**

Bacterial community growth metrics (maximum growth rate, doubling time, carrying capacity) were measured using optical density at 590 nm for bacterial communities before inoculation (day 0) and from pitcher fluid samples at the end of the experiment (day 55). Bacterial cultures and pitcher fluid samples (2 µL) were added to 198 μL R2A media in a clear flat bottom 96 well plate (53), preliminary tests found no difference in growth rates or colony forming units in R2A versus ACM so R2A was used due to cricket media sometimes being hard to take OD measurements in because of cricket particles. Plates were incubated in a plate reader (Tecan Spark) at 18 °C and absorbance was measured every 30 minutes for 48 hours. Bacterial growth metrics were analyzed using the R growthcurver package (54).

## Bacterial Community Respiration Methods

The microbial community respiration was measured (as CO_2_ produced) for both the bacterial cultures prior to the experiment (day 0) and for pitcher fluid samples at the end of the experiment (day 55), using the MicroResp system (55). Samples were added to a sterile deep well plate (250 µL) along with 250 µL acidified cricket media. The plate was fitted with a silicon mat with small holes and an indicator plate, both plates were clamped into the MicroResp apparatus and incubated in the dark at room temperature. The indicator plate contains a pH sensitive dye (cresol red 12.5 ppm, wt/wt) that shifts from magenta to yellow in the presence of carbon dioxide. The absorbance at 570 nm was measured prior to incubation, and consecutively after 6, 24, and 48 hours. Carbon dioxide concentrations were calculated using a standard curve created by incubating an indicator medium with a series of known carbon dioxide concentrations. Respiration rates were calculated for the first 24 hours of respiration as a change in the CO_2_ produced over the first 24 hours.

## Plant Photosynthesis Methods

Photosynthetic rates (µmol CO_2_ m^-2^ s^-1^) of the target pitcher (pitcher one) and secondary mature pitcher (pitcher two) were measured separately at the end of the experiment (day 55) using a LICOR-6400 photosynthesis system (LICOR Inc., Lincoln, NE) and leaf chamber fluorometer (LCF 6400-40). Measurements were taken between 1000 and 1300 h on plants removed from the growth chambers, pitchers were filled with distilled water. Photosynthetically active radiation (PAR) was set to 1200 µmol photons m^-2^ s^-1^ for 5 min. A subset of plants (three plants from each of the five treatments, n=15) had photosynthetic rates measured on the youngest mature pitcher on each plant, prior to inoculation and addition to the experiment.

The maximum quantum efficiency of photosystem II photochemistry (F_v_/F_m_)(57) was measured once daily for one week, then weekly for 8 weeks. A portable multimode chlorophyll fluorometer (Opti-Sciences) was used to measure the maximum quantum yield (F_v_/F_m_) of chlorophyll fluorescence after a 25-minute period of dark acclimation using plastic leaf clips(34). The fiber optic cable was wiped with 70% ethanol between each plant and the plastic leaf clips were sanitized with 10% bleach, rinsed with DI water, and allowed to air dry after each measurement.

## Plant Traits Used

Many plant traits were measured (leaf length, width, aperture, biomass, photosynthetic efficiency, photosynthetic rate, pitcher carbon, pitcher nitrogen, total number of leaves, new leaves). The collinearity in the response variables was assessed using a principal components analysis (PCA) on scaled data, dimension 1 explaining 77.1% of the variance and dimension 2 explaining 7.9% (Fig. S9). Along dimension 1, pitcher morphological measures (length, width, aperture, biomass) were tightly correlated with each other. Pitcher dry biomass was selected as the representative measure of plant size. Pitcher nitrogen and carbon content were selected as measures of nutrient assimilation (Fig. S9).

## ANCOM-BC

For ASV-level differential abundance explained by the treatment groups, we used *ANCOMBC* (ancombc2(data = tse, assay_name = "counts", tax_level = NULL, fix_formula = "treatment", rand_formula = "(1 | week)", p_adj_method = "fdr", pseudo_sens = TRUE, prv_cut = 0.3, group="treatment", alpha = 0.05, n_cl = 3, verbose = TRUE, global = TRUE, pairwise = TRUE)) (28). Significantly differentially abundant ASVs were visualized with box plots and a heatmap(100) by transforming the normalized reads: (log2(read count+0.5)).

## Model Structure Detail

To isolate the impact of functionally distinct bacterial communities on plant traits (after 8 weeks in planta, Questions 1 and 2), we developed Bayesian generalized linear models (GLMs) using variables based on the causal assumptions of directed acyclic graphs (DAGs). Directed acyclic graphs help identify which observed and unobserved variables to condition upon to remove non-causal associations between variables and isolate mechanisms of interest, under the assumption that the depicted pathways between variables in a research design are accurate (102, 103). Therefore, GLMs contained a variable set designed to isolate the effects of treatment and enzymatic activity from the other unspecified and unrecorded impacts of our bacterial treatments (Fig. S1) (68). Pitcher biomass was selected to represent the morphometrics and growth, along with leaf nitrogen content representing leaf nutrient acquisition. Other random effects like inoculation date and initial pitcher volume were not accounted for in the model but controlled for by the randomized design.

## Metagenomic Analyses Details

For the metagenomic analysis, the “sketch”, “gather”, and “tax annotate” functions within sourmash (version 4.8.2)(75) using kmer=31 were used to assign and quantify taxonomy using the full GTDB database (R08-RS214 403k)(76). Contigs in the coassembly and paired-end reads were binned using MAXBIN2 (2.2.7), CONCOCT (1.1.0), and Metabat2 (2.12.1), consensus bins were produced using DASTool (1.1.6)(77–80). Completeness and contamination of each metagenome assembled genome (MAG) were calculated using the presence of single-copy marker genes with CHECKM2 v.1.0.11(81). Comparisons and dereplication of MAGs employed dREP v.2.2.1(82). Taxonomy was assigned to the quality-controlled MAGs using the full GTDB database within sourmash. Bins were quality ranked according to their completeness and contamination (>90% completion, <5% redundancy), medium quality (>50% completion, <10% redundancy), low quality (<50% completion, <10% redundancy), or discarded (>10% redundancy) (83, 84). The abundance of each MAG in each treatment was calculated by first building an index for each MAG using the `index` function in bwa (85), using bwa and samtools (86) to align the individual forward and reverse sample reads to each MAG. The phylogentic tree for the MAGs was built by first extracting the marker genes from each MAG using the `ineage_wf’ and `qa’ tools from CHECKM2 (81) and then multiple sequence alignment with MAFFT (62). The phylogenetic tree was built with IQ-Tree (87) and parsed in R. Predicted gene sequences from the metatranscriptomic samples were mapped to a custom database built with the MAGs, and then used blastn (88) to quantify the number of contigs in each MAG that matched with each predicted gene.

## Metatranscriptomic Analyses Details

Differential transcript abundance was quantified using *Dream* within the *variancePartition* package (94) with treatment set as a fixed effect and plant ID set as a random intercept to account for repeated measures across three time points. The dream model was used to estimate weights using our pre-defined linear mixed model and then fit the model using the Satterthwaite approximation. The coefficient table was filtered down to those z-scores greater than or equal to the critical value of 1.645 resulting in those genes that are differentially expressed across our treatments. Within this model, contrasts were created to compare the expression levels of genes in each community treatment against the average of the other two: CommA vs (CommB + CommC)/2, CommB vs (CommA + CommC)/2, CommC vs (CommA + CommB)/2.

# **Supplementary Figures**

**
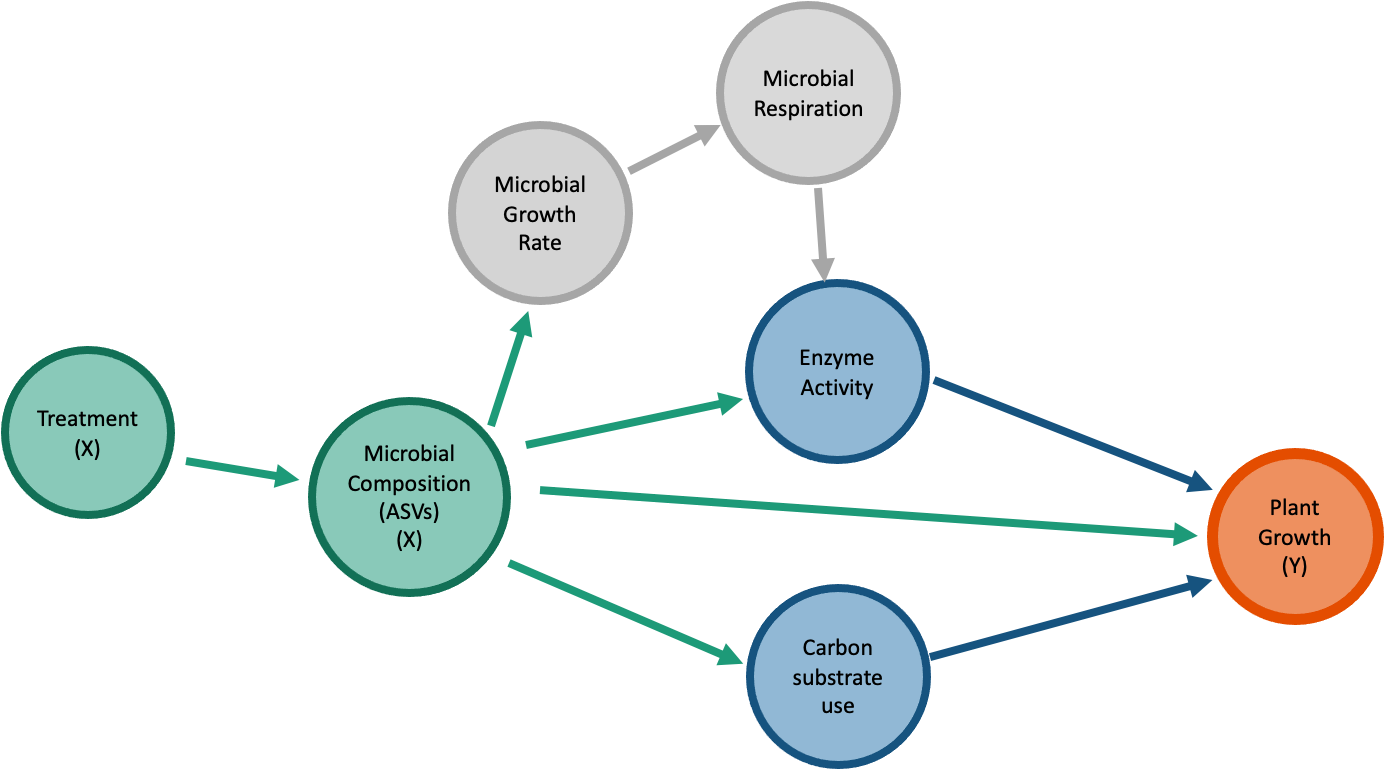
**

Figure S1. Directed Acyclic Graph (DAG) visualizing the predictor variables (microbial functions and composition) we hypothesize influence plant growth traits (biomass, leaf nitrogen content).


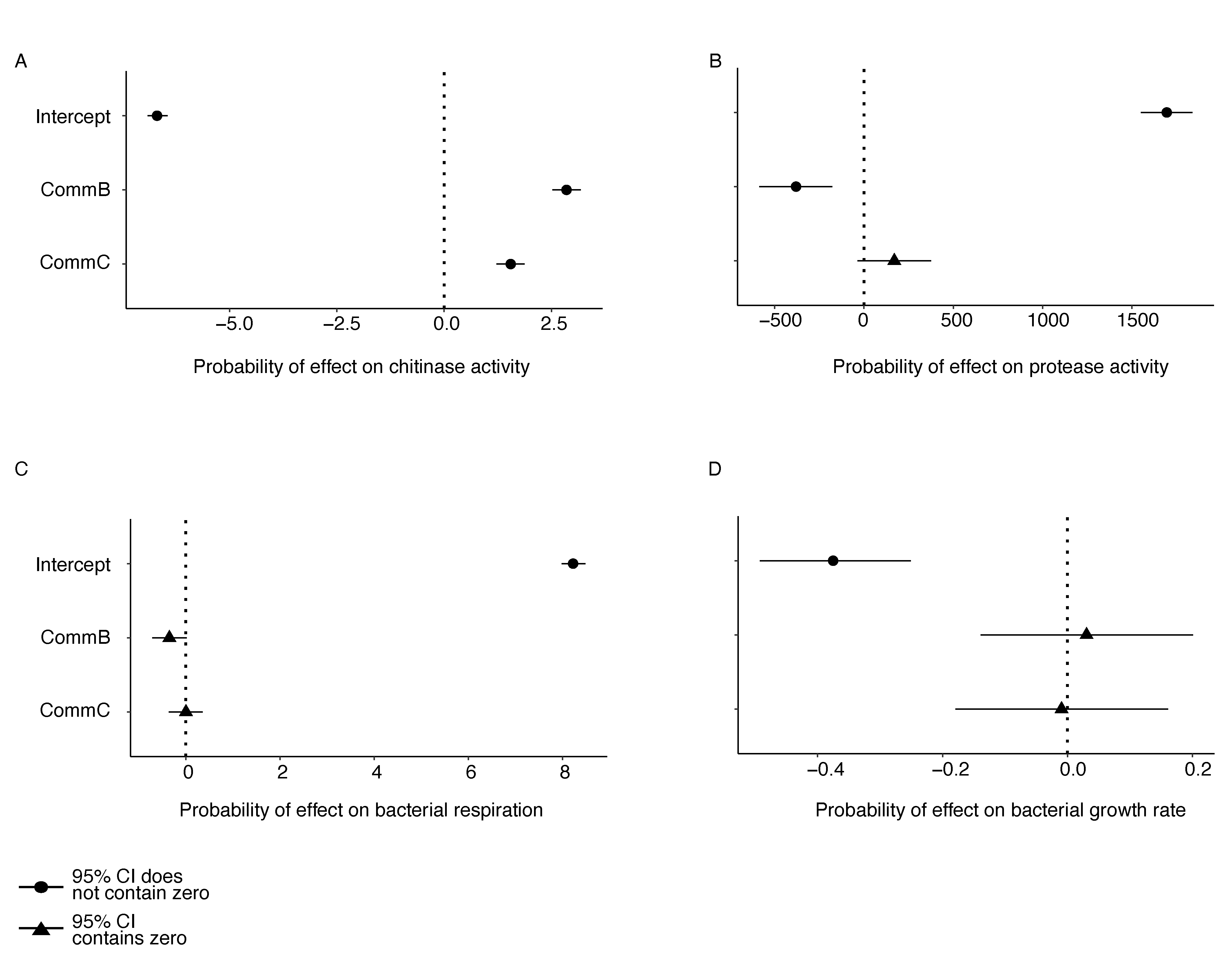


Figure S2. Functional differences in starting bacterial communities (before inoculation). Parameter estimates for the probability of differences between bacterial community treatments on (**A**) chitinase activity, (**B**) protease activity, (**C**) bacterial respiration of bacterial community culture, and (**D**) bacterial growth rate before the start of the experiment based on generalized linear models (function ~ treatment). Black points indicate median parameter estimates with associated 95% credibility intervals. Parameters with 95% credibility intervals that did not include zero (dashed vertical line) were considered nonzero effects on the response (circles vs. triangles). CommA is set as the baseline (vertical dashed line) predictor in all models containing treatment.


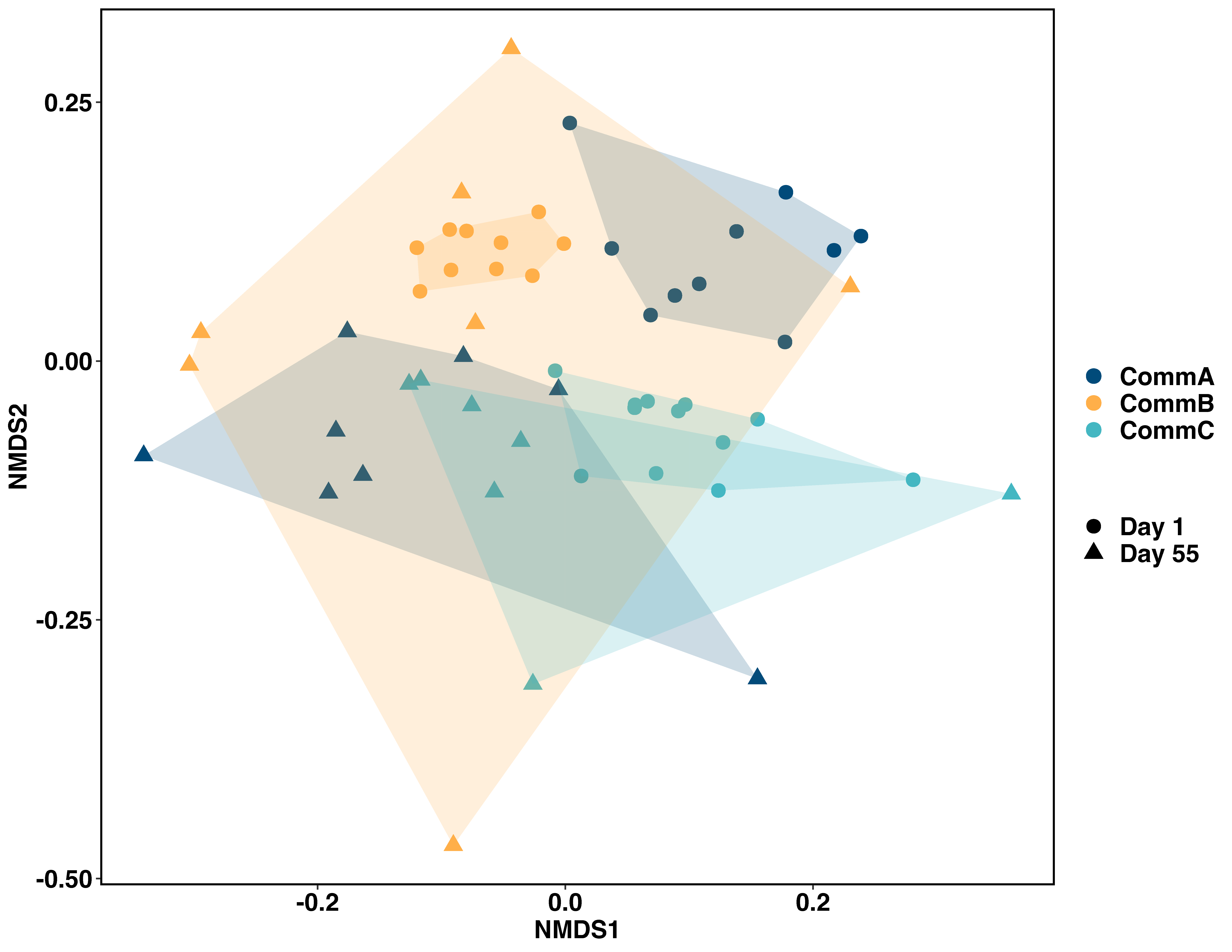


Figure S3. Non-metric multidimensional scaling (NMDS) based on Bray Curtis dissimilarity from Biolog EcoPlate physiological profiles for pitcher bacterial communities at day 1 and day 55. Treatments are coded by color and time by shape. Significant (p<0.05) differences between treatment groups within individual time points and between day 1 and 55 (Table S1).

**
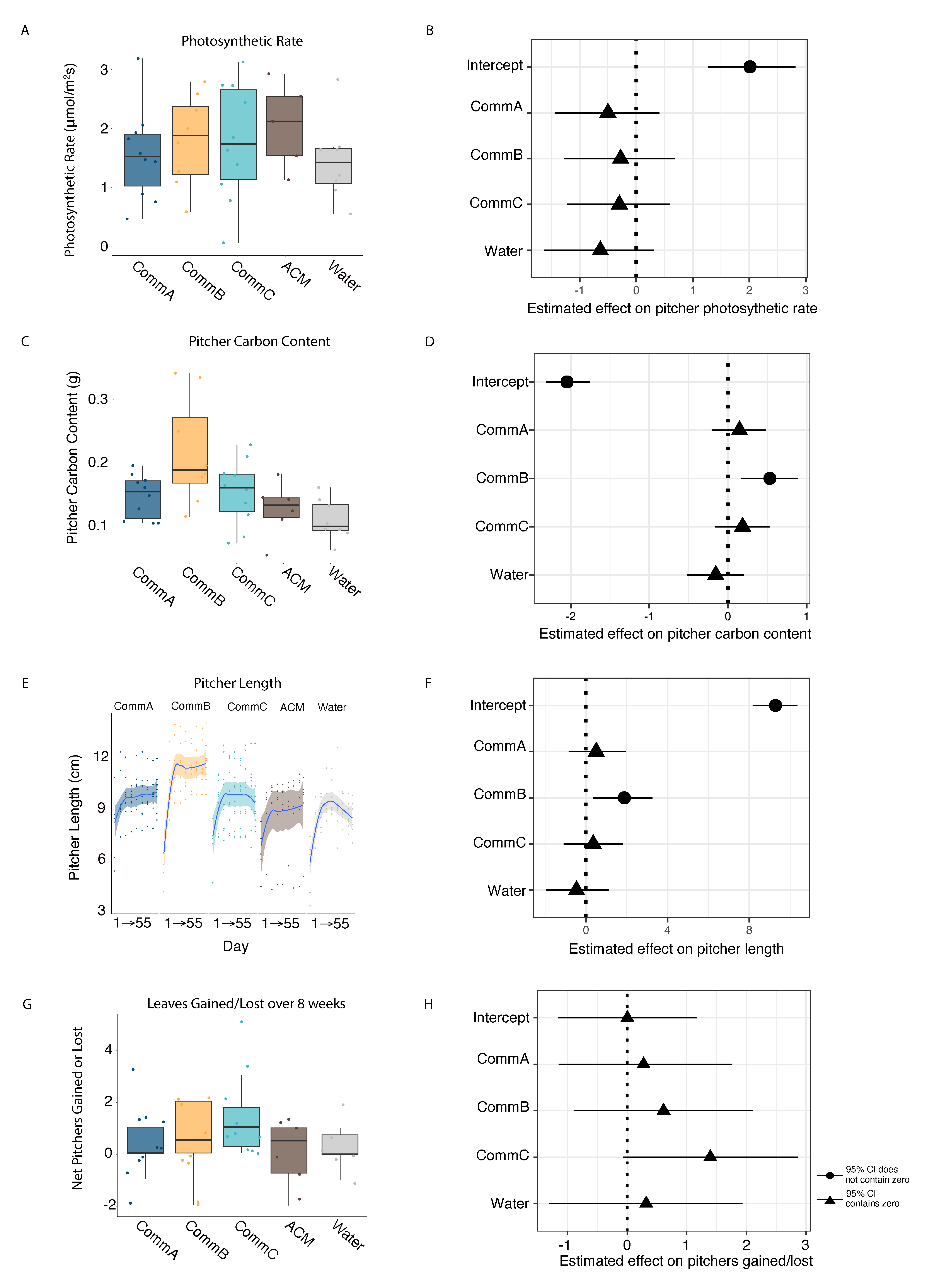
**

Figure S4. Exploring the effect of treatment on other plant traits. (**A**) The photosynthetic rate (µmol CO_2_ m^-2^ s^-1^) was measured on the treated pitcher at the end of the experiment (day 55). (**B**) Posterior parameter estimates for the effects of treatment on pitcher photosynthetic rate. (**C**) Pitcher carbon content was measured at the end of the experiment; carbon content was higher in CommB compared to the experimental and control treatments. (**D**) Posterior parameter estimates for the effects of treatment on pitcher carbon content. (**E**) Pitcher length was measured weekly for each treated pitcher; pitcher length was higher in CommB compared to the experimental and control treatments and correlated with other plant morphology measures. (**F**) Posterior parameter estimates for the effects of treatment on pitcher length. (**G**) Number of net pitchers gained or lost (starting number of pitchers minus ending number of pitchers) across all treatments. (**H**) Posterior parameter estimates for the effects of treatment on net pitchers gained or lost. **(B, D, F, H)** Symbols represent the median parameter estimates and lines represent the 95% credible intervals for the parameter estimate. Parameters with 95% credibility intervals that did not include zero (dashed vertical line) were considered nonzero effects on the response (circles vs. triangles), ACM with no bacterial additions is set as the baseline predictor (vertical dashed line).


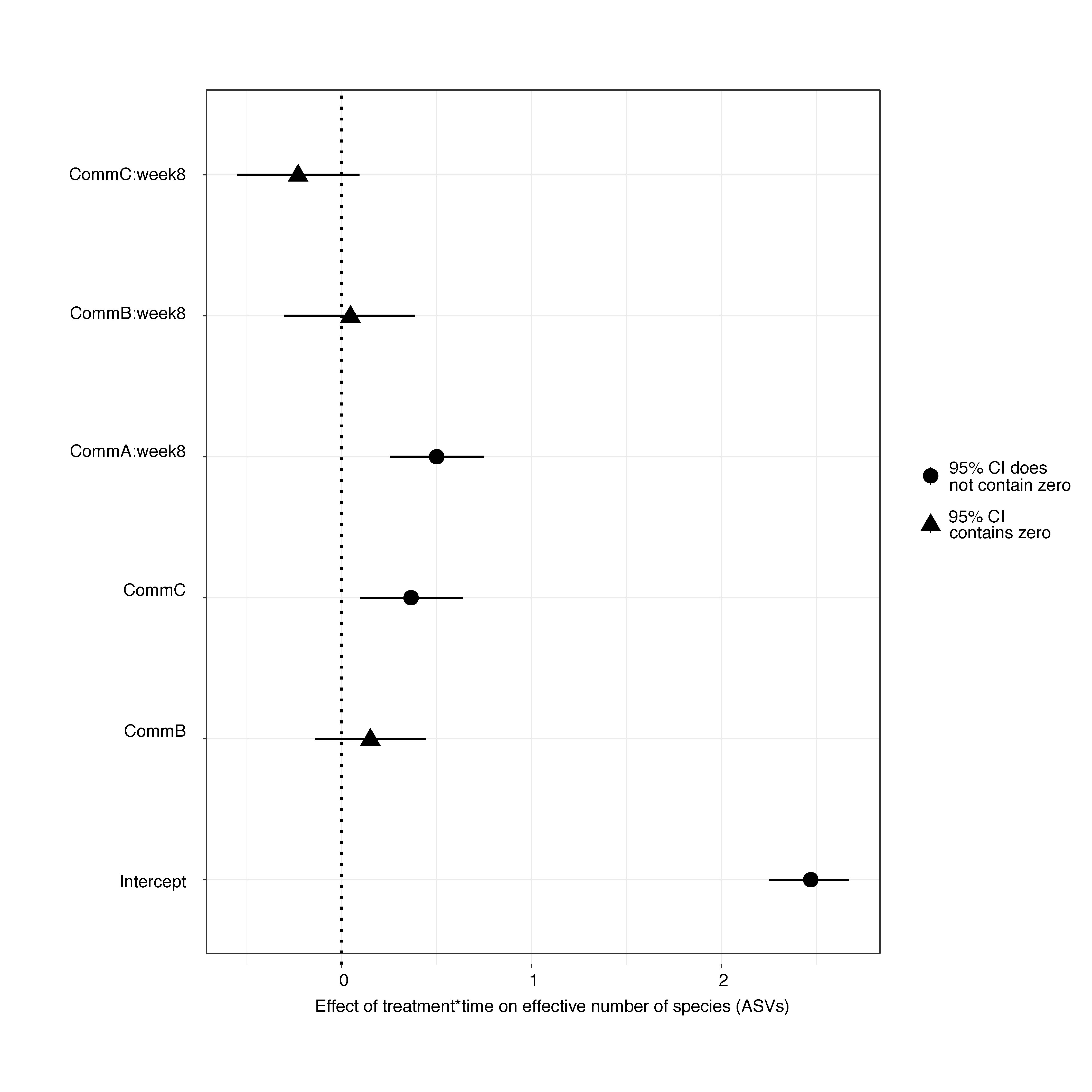


Figure S5. Differences in alpha diversity between bacterial communities. Posterior parameter estimates for the effects of treatment and time on the effective number of species (ASVs) based on generalized linear model. Symbols represent the median parameter estimates and lines represent the 95% credible intervals for the parameter estimate. Parameters with 95% credibility intervals that did not include zero (dashed vertical line) were considered nonzero effects on the response (circles vs. triangles), CommA and week 1 are set as the baseline predictor (see previous figures).

**
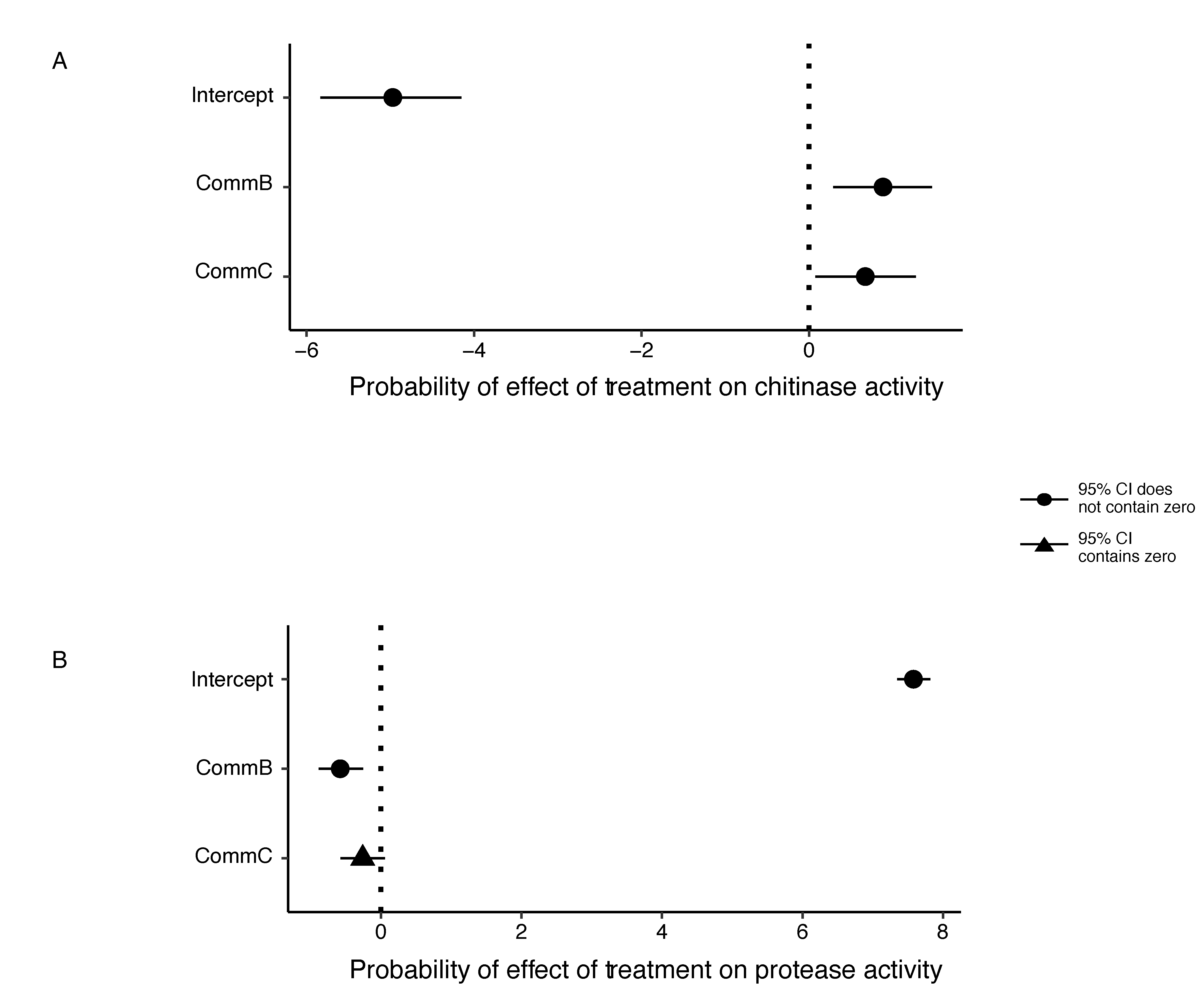
**

Figure S6. Posterior parameter estimates for the effects of treatment on chitinase and protease activity. Subset to just those plants whose communities were sequenced for metatranscriptomics (3 plants per treatment). Symbols represent the median parameter estimates and lines represent the 95% credible intervals for the parameter estimate. Parameters with 95% credibility intervals that did not include zero (dashed vertical line) were considered nonzero effects on the response (circles vs. triangles). CommA was set as the baseline predictor and time was included in the model as a random intercept. CommB and CommC had higher chitinase activity compared to CommA; CommB had higher protease activity than both CommA and CommC.


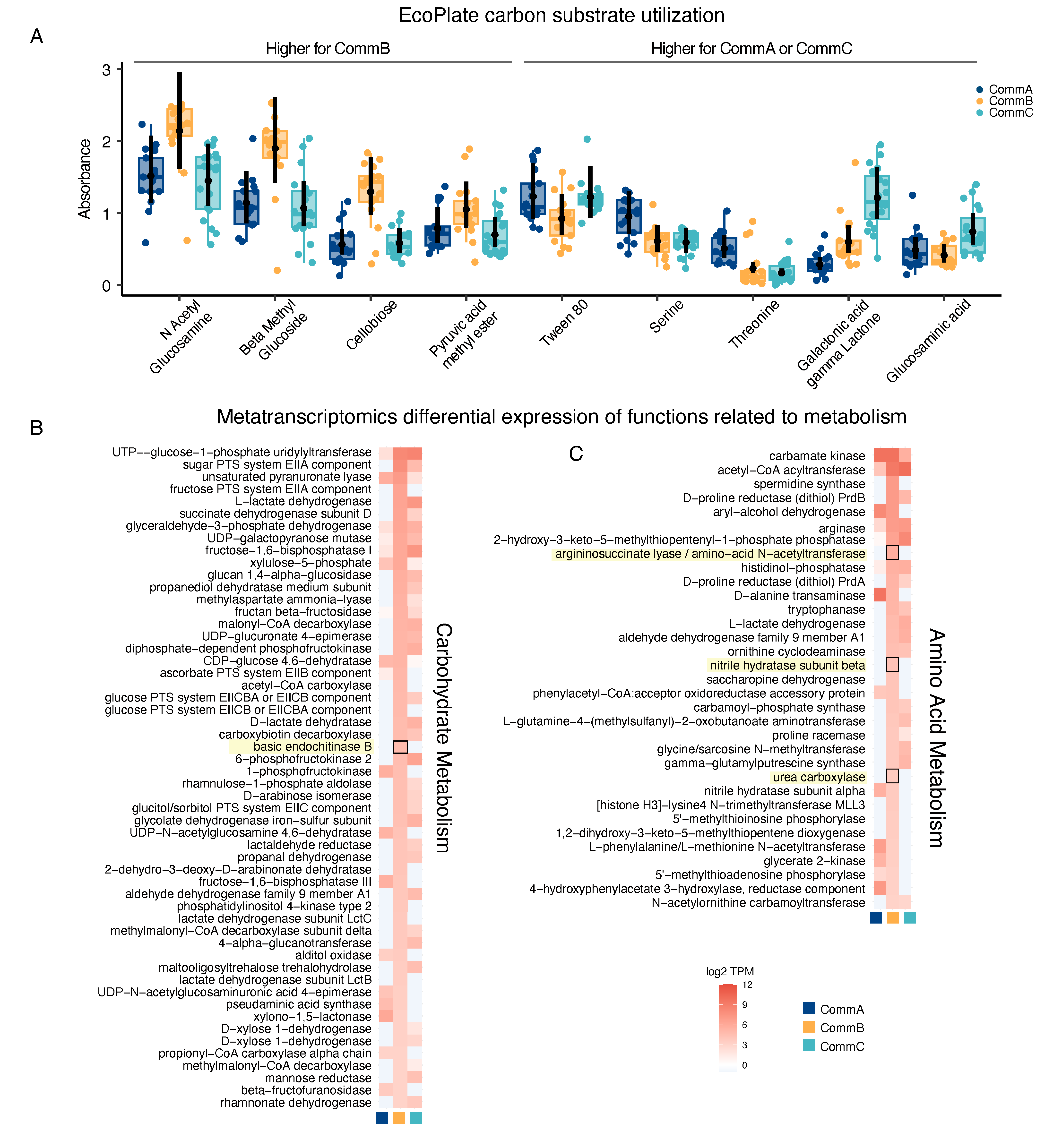


Figure S7. Differences in EcoPlate carbon substrate metabolism among the three treatments. (**A**) A subset of nine of the 31 carbon substrates which showed differences in absorbance among samples of the three treatments, showing differential capacity to use these substrates. Points are colored by treatment and represent the absorbance for an individual sample for that substrate, day 1 and day 55 samples combined for each substrate. Box plots and colored points represent the raw data, colored whiskers representing data 1.5 times the interquartile range. The black points represent the median marginal effects from the model testing the effect of treatment and substrate on absorbance with week as a random intercept, the black vertical bars represent the 95% credibility intervals around each estimate. Normalized abundance of KOs associated with (**B**) carbohydrate metabolism and (**C**) amino acid metabolism, using significantly differentially abundant genes summed according to KO function. Heat maps are grouped by treatment and transcripts per million (TPM) are transformed (log2(TPM+0.5)). This subset of KO functions show instances where expression in CommB was significantly higher (by at least 4 log2(TPM+0.5)) than either CommA or CommC. Functions that are particularly relevant for this study are highlighted in yellow.

**
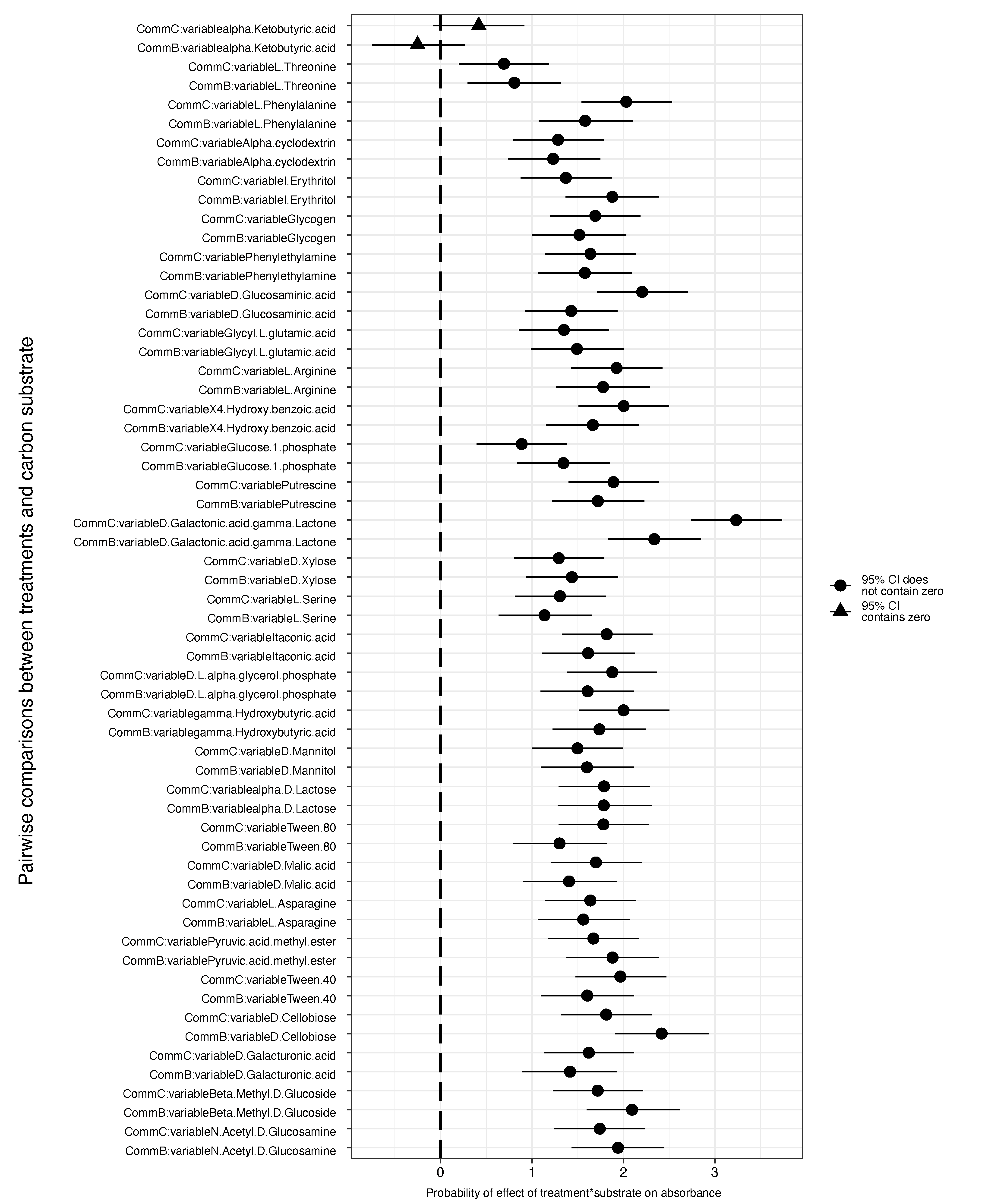
**

Figure S8. Posterior parameter estimates for the effects of treatment and substrate on absorbance. Symbols represent the median parameter estimates and lines represent the 95% Cis for the parameter estimate. Parameters with 95% credibility intervals that did not include zero (dashed vertical line) were considered nonzero effects on the response (circles vs. triangles). CommA and carbon substrate X2.Hydroxy.benzoic.acid were set as the baseline predictors (vertical dashed line).


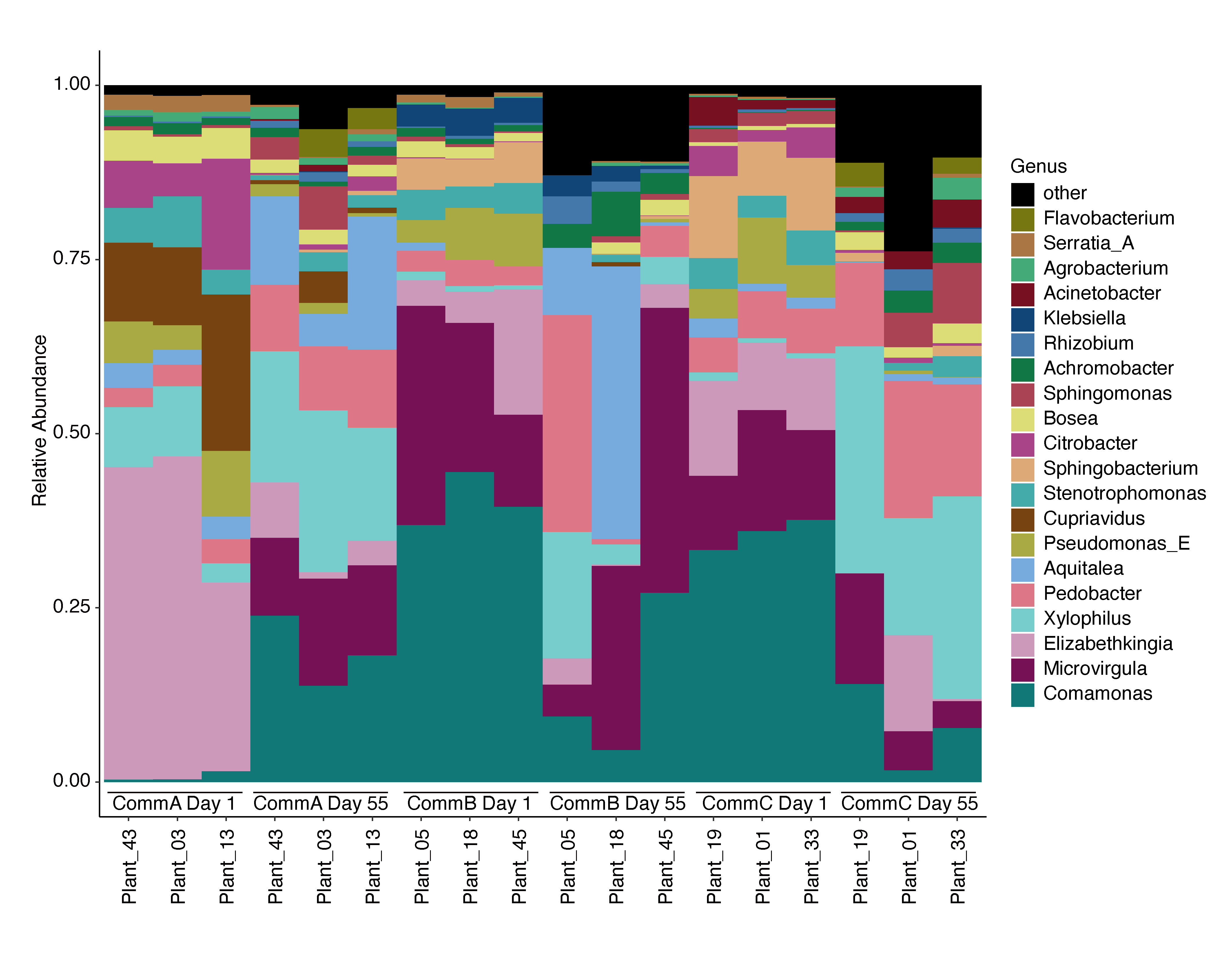


Figure S9. Bacterial relative abundance based on metagenomic analysis. Taxonomic composition differences between the three microbial community treatments identified from shotgun metagenomic analysis of pitcher fluid samples. Relative abundance to the top 20 most abundant genera at day 1 and day 55 for each of the three bacterial community treatments. Taxonomy assigned using the GTDB database based on kmer=31.

**
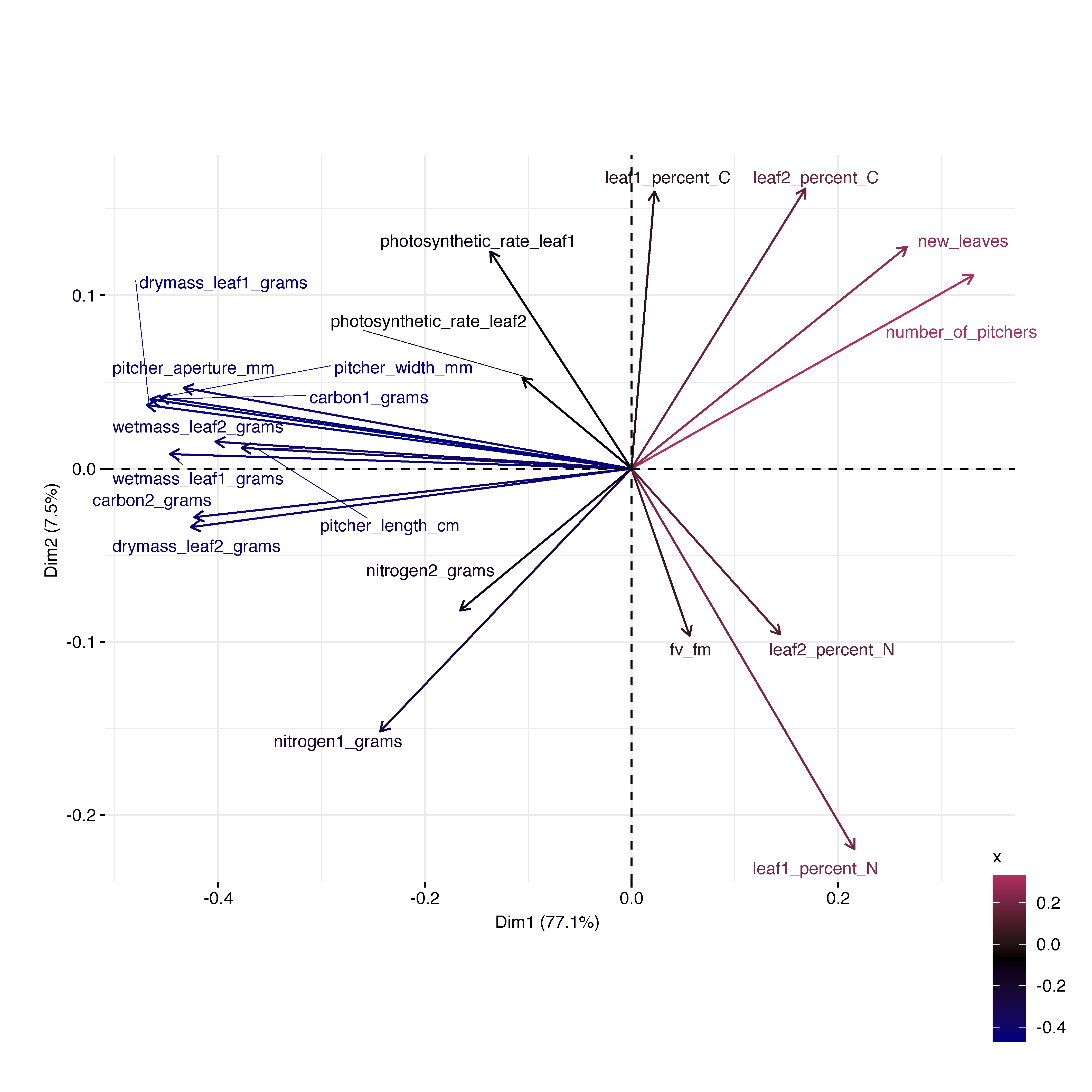
**

Figure S10. Correlation of plant traits. Principal components analysis (PCA) of scaled plant traits measured for each pitcher (target pitcher = leaf1 and the next youngest pitcher = leaf2 on each plant). The color of the arrows represents the correlation with Dimension (Dim) 1 (x-axis). Dimension 1 explains 77.1% of the variance and dimension 2 explains 7.9% for a total of 85% explained. Along dimension 1, pitcher morphological measures (length, width, aperture, biomass) were tightly correlated with each other so pitcher biomass (grams) was selected to represent plant size and pitcher nitrogen content (mg) represented plant traits that varied along dimension 2 and did not strongly correlate.

# **Supplementary Tables**

Table S1. Pairwise comparisons in Biolog EcoPlate physiological profiles at day 1 and 55 for pitcher bacterial communities (CommA, CommB, CommC), plates read after 72 hours.

com5 ~ treatment * day, strata = plant, permutations 999

| CommC vs. CommA |  | Df | F | Pr(>F) |
| --- | --- | --- | --- | --- |
|  | Treatment | 1 | 9.9688 | 0.001 |
|  | Day | 1 | 10.0143 | 0.001 |
|  | Treatment:Day | 1 | 4.0190 | 0.014 |
| CommC vs. CommB |  |  |  |  |
|  | Treatment | 1 | 9.8677 | 0.001 |
|  | Day | 1 | 6.7874 | 0.002 |
|  | Treatment:Day | 1 | 2.0407 | 0.090 |
| CommA vs. CommB |  |  |  |  |
|  | Treatment | 1 | 7.8048 | 0.004 |
|  | Day | 1 | 6.7125 | 0.004 |
|  | Treatment:Day | 1 | 3.5927 | 0.023 |

Table S2. Unweighted UniFrac pairwise comparisons based on 16S amplicon sequencing. Using pairwise.adonis2(uu.dist.16s.treat ~ treatment + day, strata = Null, permutations 999)

| CommC vs. CommA |  | Df | R^2^ | F | Pr(>F) |
| --- | --- | --- | --- | --- | --- |
|  | Treatment | 1 | 0.27025 | 61.2079 | 0.001 |
|  | Day | 7 | 0.08510 | 2.7535 | 0.001 |
| CommC vs. CommB |  |  |  |  |  |
|  | Treatment | 1 | 0.14483 | 26.7313 | 0.001 |
|  | Day | 7 | 0.14542 | 3.8345 | 0.001 |
| CommA vs. CommB |  |  |  |  |  |
|  | Treatment | 1 | 0.26353 | 53.9211 | 0.001 |
|  | Day | 7 | 0.10113 | 2.9559 | 0.001 |

Table S3. Using analysis of compositions of microbiomes with bias correction (ANCOM-BC), we identified 38 differentially abundant taxa between our treatments and plotted these estimates along with their estimated error**.** lfc=log fold change, DA=differentially abundant, se=standard error. Ancombc2(data = df, assay_name = "counts", tax_level = NULL, fix_formula = "treatment", rand_formula = "(1 | day)", p_adj_method = "fdr", prv_cut = 0.3, group="treatment", alpha = 0.05, global = TRUE, pairwise = TRUE)

| ASV | lfc_CommA | lfc_CommC | se_CommA | se_CommC | DA_CommA | DA_CommC |
| --- | --- | --- | --- | --- | --- | --- |
| ASV18 | 2.28088287 | 0 | 0.85764661 | 0.39151026 | TRUE | FALSE |
| ASV2 | 0.85647721 | 0.75524822 | 0.3262171 | 0.33665869 | TRUE | FALSE |
| ASV28 | -0.6789646 | -0.4036594 | 0.23550739 | 0.2442019 | TRUE | FALSE |
| ASV65 | -1.3745555 | 0 | 0.48727129 | 0.39151026 | TRUE | FALSE |
| ASV4 | 0.82878776 | 0.71886 | 0.22909797 | 0.23958411 | TRUE | TRUE |
| ASV9 | 0.07055617 | -0.7617091 | 0.23875579 | 0.25316065 | FALSE | TRUE |
| ASV19 | -2.9715466 | -0.5553903 | 0.79710055 | 0.23307774 | TRUE | FALSE |
| ASV1 | -0.8875793 | -0.1959028 | 0.30478657 | 0.30676597 | TRUE | FALSE |
| ASV11 | 0.4710853 | 3.7877542 | 0.45936395 | 0.432524 | FALSE | TRUE |
| ASV38 | 2.33324976 | 2.64994413 | 0.46725961 | 0.4747444 | TRUE | TRUE |
| ASV34 | 0 | -1.1127227 | 0.38320487 | 0.39420829 | FALSE | TRUE |
| ASV12 | -2.3953791 | -0.1139894 | 0.30102857 | 0.29387924 | TRUE | FALSE |
| ASV8 | -1.1316007 | -0.7806544 | 0.27316502 | 0.28154805 | TRUE | TRUE |
| ASV15 | -0.1816149 | 2.51234793 | 0.31793415 | 0.27634194 | FALSE | TRUE |
| ASV84 | -0.8745872 | -0.0362883 | 0.27831542 | 0.25077011 | TRUE | FALSE |
| ASV57 | -0.9444645 | -0.0494317 | 0.2862049 | 0.24735335 | TRUE | FALSE |
| ASV106 | -0.8664169 | -0.078243 | 0.26386658 | 0.22958842 | TRUE | FALSE |
| ASV67 | 1.26668178 | 2.33520807 | 0.32291121 | 0.31662338 | TRUE | TRUE |
| ASV21 | 0.20373376 | -0.8973839 | 0.28504718 | 0.29655565 | FALSE | TRUE |
| ASV59 | -0.7356318 | -1.0637647 | 0.22144103 | 0.23245335 | TRUE | TRUE |
| ASV5 | 1.01782947 | -0.5900468 | 0.25855364 | 0.26786661 | TRUE | FALSE |
| ASV6 | -1.0924291 | -0.7822658 | 0.23655704 | 0.23814965 | TRUE | TRUE |
| ASV31 | -1.967328 | -1.3175785 | 0.32111856 | 0.27845587 | TRUE | TRUE |
| ASV30 | 0.02063799 | -1.1920085 | 0.26247751 | 0.27151989 | FALSE | TRUE |
| ASV88 | -1.2316125 | -1.1584666 | 0.37293824 | 0.31068293 | TRUE | TRUE |
| ASV10 | 2.48587669 | 3.87683718 | 0.77798985 | 0.70223672 | TRUE | TRUE |
| ASV121 | -1.5892854 | -0.1279019 | 0.30353483 | 0.23251559 | TRUE | FALSE |
| ASV69 | -0.7396856 | -1.5488697 | 0.20033138 | 0.21402957 | TRUE | TRUE |
| ASV90 | -1.0598345 | -0.3681058 | 0.24679575 | 0.25811064 | TRUE | FALSE |
| ASV123 | -0.895807 | -0.1304857 | 0.36140543 | 0.27778054 | TRUE | FALSE |
| ASV85 | -2.5631267 | -0.697296 | 0.38623488 | 0.28440992 | TRUE | FALSE |
| ASV32 | -2.6922573 | 0.32432347 | 0.32897822 | 0.23346745 | TRUE | FALSE |
| ASV41 | -1.7207563 | 0.77619731 | 0.72608583 | 0.30248531 | TRUE | TRUE |
| ASV62 | -2.1179012 | -0.3840785 | 0.42798943 | 0.26251152 | TRUE | FALSE |
| ASV35 | -1.3413746 | 0.42010938 | 0.30682567 | 0.27697165 | TRUE | FALSE |
| ASV58 | -1.6998289 | 0.8386147 | 0.42003553 | 0.27163856 | TRUE | TRUE |
| ASV46 | -1.0877948 | 0.00138532 | 0.35357518 | 0.34144047 | TRUE | FALSE |
| ASV39 | -0.6185634 | 0.25422089 | 0.2425294 | 0.3216076 | TRUE | FALSE |

Table S4. MAG taxonomy and quality metrics.

| **Name** | **new_bin_name** | **Completeness** | **Contamination** | **quality_grade** | **Contig_N50** | **Average_Gene_Length** | **Genome_Size** | **GC_Content** | **Total_Coding_Sequences** | **lineage** | **query_md5** | **f_weighted_at_rank** | **bp_match_at_rank** | **query_ani_at_rank** |
| --- | --- | --- | --- | --- | --- | --- | --- | --- | --- | --- | --- | --- | --- | --- |
| maxbin.41 | MAG_34 | 99.99 | 10.05 | discard | 117302 | 304.1812541 | 6320792 | 0.62 | 6124 | d__Bacteria; p__Pseudomonadota; c__Gammaproteobacteria;o__Burkholderiales;f__Burkholderiaceae_B;g__Comamonas;s__Comamonas testosteroni_C | 2503fe2d | 0.245283019 | 1560000 | 0.897695969 |
| concoct.81_sub | MAG_27 | 90.82 | 10.74 | discard | 10693 | 276.216608 | 6750378 | 0.6 | 7105 | d__Bacteria; p__Pseudomonadota; c__Alphaproteobacteria;o__Rhizobiales;f__Rhizobiaceae;g__Agrobacterium;s__Agrobacterium fabacearum | 7ea57366 | 0.10521701 | 720000 | 0.996175442 |
| concoct.74 | MAG_44 | 86.49 | 11.21 | discard | 4326 | 256.3423595 | 5575643 | 0.54 | 6315 | d__Bacteria; p__Pseudomonadota; c__Gammaproteobacteria;o__Enterobacterales;f__Enterobacteriaceae;g__Serratia_A;s__Serratia_A fonticola | 62bbd086 | 0.943229836 | 5134000 | 0.871970498 |
| concoct.70_sub | MAG_11 | 73.94 | 21.93 | discard | 2301 | 228.6382245 | 3372305 | 0.46 | 4348 | d__Bacteria; p__Bacillota_C; c__Negativicutes;o__Sporomusales_C;f__DSM-15969;g__Anaerospora;s__Anaerospora sp002337925 | 900253b4 | 0.032017408 | 103000 | 0.93483343 |
| metabat2.44_sub | MAG_47 | 88.45 | 35.8 | discard | 5952 | 301.0039273 | 6906112 | 0.68 | 6875 | d__Bacteria; p__Pseudomonadota; c__Gammaproteobacteria;o__Xanthomonadales;f__Rhodanobacteraceae;g__Dokdonella_A;s__Dokdonella_A sp017744955 | 2a7724f1 | 0.014307229 | 95000 | 0.859142103 |
| concoct.3 | MAG_19 | 100 | 37.58 | discard | 137612 | 339.2890728 | 8413010 | 0.41 | 7614 | d__Bacteria; p__Bacteroidota; c__Bacteroidia;o__Sphingobacteriales;f__Sphingobacteriaceae;g__Pedobacter;s__Pedobacter nutrimenti | 6b7d278f | 0.409965348 | 3431000 | 0.822039738 |
| concoct.84 | MAG_48 | 99.82 | 73.45 | discard | 40959 | 328.1228387 | 8471315 | 0.67 | 7750 | d__Bacteria; p__Pseudomonadota; c__Gammaproteobacteria;o__Xanthomonadales;f__Xanthomonadaceae;g__Stenotrophomonas;s__Stenotrophomonas sp002192255 | 26e87d98 | 0.088797302 | 711000 | 0.829564005 |
| metabat2.12 | MAG_45 | 100 | 0.04 | high | 269072 | 312.4638309 | 3919568 | 0.39 | 3691 | d__Bacteria; p__Pseudomonadota; c__Gammaproteobacteria;o__Pseudomonadales;f__Moraxellaceae;g__Acinetobacter;s__Acinetobacter seifertii | 12354c55 | 0.88215998 | 3496000 | 0.953139318 |
| maxbin.11 | MAG_01 | 100 | 0.06 | high | 74848 | 323.2122769 | 3887567 | 0.71 | 3698 | d__Bacteria; p__Actinomycetota; c__Actinomycetia;o__Actinomycetales;f__Microbacteriaceae;g__Leifsonia;s__Leifsonia sp001898805 | 5a696026 | 0.116290984 | 454000 | 0.901116999 |
| concoct.92_sub | MAG_16 | 95.5 | 0.13 | high | 42866 | 333.9214864 | 3678956 | 0.37 | 3337 | d__Bacteria; p__Bacteroidota; c__Bacteroidia;o__Flavobacteriales;f__Flavobacteriaceae;g__Flavobacterium;s__Flavobacterium microcysteis | 1f669bfb | 0.128183832 | 463000 | 0.890346875 |
| metabat2.91 | MAG_02 | 99.77 | 0.16 | high | 336160 | 326.6919406 | 1988347 | 0.63 | 1886 | d__Bacteria; p__Actinomycetota; c__Actinomycetia;o__Actinomycetales;f__Microbacteriaceae;g__Leifsonia;s__Leifsonia sp009765225 | e99fa88c | 0.00305033 | 6000 | 0.989066875 |
| maxbin.10 | MAG_09 | 98.42 | 0.35 | high | 45578 | 304.6598456 | 2732790 | 0.46 | 2590 | d__Bacteria; p__Bacillota_A; c__Clostridia;o__Oscillospirales;f__Acutalibacteraceae;g__Caproiciproducens;s__Caproiciproducens sp002338255 | a858386e | 0.00795756 | 21000 | 0.875708865 |
| concoct.7 | MAG_39 | 100 | 0.53 | high | 37177 | 317.9608681 | 6190281 | 0.67 | 5852 | d__Bacteria; p__Pseudomonadota; c__Gammaproteobacteria;o__Burkholderiales;f__Burkholderiaceae_C;g__Achromobacter;s__Achromobacter sp016428805 | 41529c77 | 0.877144707 | 5419000 | 0.971645788 |
| concoct.20 | MAG_22 | 90.9 | 0.64 | high | 69468 | 341.2928881 | 4401942 | 0.44 | 3923 | d__Bacteria; p__Bacteroidota; c__Bacteroidia;o__Sphingobacteriales;f__Sphingobacteriaceae;g__Pedobacter;s__Pedobacter sp016429155 | c2f378b7 | 0.711205929 | 3167000 | 0.846410758 |
| metabat2.80 | MAG_14 | 98.85 | 0.68 | high | 149806 | 341.1593153 | 5297186 | 0.46 | 4557 | d__Bacteria; p__Bacteroidota; c__Bacteroidia;o__Chitinophagales;f__Chitinophagaceae;g__Edaphocola;s__Edaphocola koreensis | 81013bce | 0.002299732 | 12000 | 0.99840909 |
| concoct.41 | MAG_17 | 100 | 0.99 | high | 381815 | 322.5015819 | 4452723 | 0.36 | 4109 | d__Bacteria; p__Bacteroidota;c__Bacteroidia;o__Flavobacteriales;f__Weeksellaceae;g__Elizabethkingia;s__Elizabethkingia miricola | a60dd1d3 | 0.975498054 | 4260000 | 0.996899806 |
| concoct.48_sub | MAG_23 | 100 | 1.05 | high | 640265 | 343.4683262 | 6769677 | 0.39 | 5825 | d__Bacteria; p__Bacteroidota;c__Bacteroidia;o__Sphingobacteriales;f__Sphingobacteriaceae;g__Sphingobacterium;s__Sphingobacterium siyangense | 8ae0385f | 0.449198647 | 3055000 | 0.999200091 |
| concoct.60 | MAG_31 | 92.66 | 1.22 | high | 22989 | 306.3097527 | 4429949 | 0.68 | 4368 | d__Bacteria; p__Pseudomonadota;c__Alphaproteobacteria;o__Sphingomonadales;f__Sphingomonadaceae;g__Sphingomonas;s__Sphingomonas sp017418975 | 95e86b8b | 0.712693358 | 3133000 | 0.857637285 |
| concoct.83 | MAG_26 | 92.42 | 1.22 | high | 174707 | 315.8816997 | 4397229 | 0.59 | 4142 | d__Bacteria; p__Pseudomonadota;c__Alphaproteobacteria;o__Rhizobiales;f__Rhizobiaceae;g__Agrobacterium;s__Agrobacterium fabacearum | 9e7e1912 | 0.900923215 | 4001000 | 0.97451457 |
| concoct.82_sub | MAG_21 | 99.88 | 1.3 | high | 672634 | 349.3753887 | 5332767 | 0.39 | 4502 | d__Bacteria; p__Bacteroidota;c__Bacteroidia;o__Sphingobacteriales;f__Sphingobacteriaceae;g__Pedobacter;s__Pedobacter sp016429065 | 65ea6890 | 0.978470676 | 5272000 | 0.947240999 |
| maxbin.12_sub | MAG_10 | 100 | 1.63 | high | 332405 | 308.1145038 | 4483247 | 0.42 | 4192 | d__Bacteria; p__Bacillota_C;c__Negativicutes;o__Propionisporales;f__DSM-13327;g__Pelosinus;s__Pelosinus fermentans_A | be376984 | 0.005874379 | 26000 | 0.992992627 |
| metabat2.65 | MAG_04 | 99.82 | 1.64 | high | 142208 | 324.0040777 | 4514432 | 0.64 | 4169 | d__Bacteria; p__Actinomycetota;c__Actinomycetia;o__Mycobacteriales;f__Mycobacteriaceae;g__Gordonia;s__Gordonia polyisoprenivorans | 5ee9f234 | 0.009036808 | 41000 | 0.991881831 |
| concoct.78 | MAG_24 | 98.96 | 1.99 | high | 15182 | 289.8501554 | 4635018 | 0.68 | 4825 | d__Bacteria; p__Pseudomonadota;c__Alphaproteobacteria;o__Caulobacterales;f__Caulobacteraceae;g__Phenylobacterium;s__Phenylobacterium sp013822795 | e62569ec | 0.059270517 | 273000 | 0.894925021 |
| maxbin.54 | MAG_18 | 99.89 | 2.02 | high | 208541 | 326.176505 | 3690367 | 0.34 | 3422 | d__Bacteria; p__Bacteroidota;c__Bacteroidia;o__Flavobacteriales;f__Weeksellaceae;g__Epilithonimonas;s__Epilithonimonas hispanica | ee598531 | 0.02406344 | 88000 | 0.998116444 |
| concoct.77_sub | MAG_15 | 95.43 | 2.03 | high | 19859 | 327.5583248 | 5424429 | 0.34 | 4895 | d__Bacteria; p__Bacteroidota;c__Bacteroidia;o__Flavobacteriales;f__Flavobacteriaceae;g__Flavobacterium;s__Flavobacterium chilense | b41e366b | 0.968813811 | 5219000 | 0.998978495 |
| concoct.35 | MAG_43 | 100 | 2.04 | high | 173044 | 314.8735437 | 5020425 | 0.52 | 4721 | d__Bacteria; p__Pseudomonadota;c__Gammaproteobacteria;o__Enterobacterales;f__Enterobacteriaceae;g__Citrobacter;s__Citrobacter braakii | 0f1b4e11 | 0.951840796 | 4783000 | 0.912880966 |
| maxbin.21 | MAG_36 | 92.78 | 2.81 | high | 274989 | 317.8978723 | 3584135 | 0.47 | 3525 | d__Bacteria; p__Pseudomonadota;c__Gammaproteobacteria;o__Burkholderiales;f__Burkholderiaceae_B;g__Variovorax;s__Variovorax sp003019815 | 0e6d1763 | 0.001114827 | 4000 | 0.929938837 |
| concoct.58 | MAG_25 | 93.07 | 3.21 | high | 8686 | 279.935212 | 3619376 | 0.69 | 3797 | d__Bacteria; p__Pseudomonadota;c__Alphaproteobacteria;o__Rhizobiales;f__Ancalomicrobiaceae;g__Pinisolibacter;s__Pinisolibacter sp002298965 | aef23108 | 0.186326702 | 665000 | 0.999298167 |
| concoct.95 | MAG_46 | 100 | 3.44 | high | 27401 | 310.4301915 | 7101217 | 0.63 | 6790 | d__Bacteria; p__Pseudomonadota;c__Gammaproteobacteria;o__Pseudomonadales;f__Pseudomonadaceae;g__Pseudomonas_E;s__Pseudomonas_E fluorescens_AP | f14046b1 | 0.814279703 | 5805000 | 0.996640004 |
| concoct.69 | MAG_42 | 99.93 | 3.45 | high | 99600 | 321.2930811 | 4189321 | 0.61 | 3859 | d__Bacteria; p__Pseudomonadota;c__Gammaproteobacteria;o__Burkholderiales;f__Chromobacteriaceae;g__Aquitalea;s__Aquitalea magnusonii | 5a4d1c90 | 0.776708373 | 3228000 | 0.924863014 |
| metabat2.16_sub | MAG_29 | 92.53 | 3.82 | high | 150427 | 313.0756176 | 4860522 | 0.62 | 4655 | d__Bacteria; p__Pseudomonadota;c__Alphaproteobacteria;o__Rhizobiales;f__Rhizobiaceae;g__Shinella;s__Shinella sumterensis | 9efb1a37 | 0.035237698 | 169000 | 0.99339445 |
| concoct.38 | MAG_38 | 97.02 | 4.1 | high | 28539 | 313.8317801 | 4416768 | 0.69 | 4292 | d__Bacteria; p__Pseudomonadota;c__Gammaproteobacteria;o__Burkholderiales;f__Burkholderiaceae_B;g__Xylophilus;s__Xylophilus sp016428875 | 3e768fb3 | 0.908232119 | 4038000 | 0.932945607 |
| concoct.113 | MAG_28 | 100 | 4.12 | high | 227871 | 337.2025518 | 4202342 | 0.61 | 3762 | d__Bacteria; p__Pseudomonadota;c__Alphaproteobacteria;o__Rhizobiales;f__Rhizobiaceae;g__Agrobacterium;s__Agrobacterium fabacearum | 8c56278f | 0.027310924 | 117000 | 0.847287951 |
| concoct.46 | MAG_20 | 66.93 | 0.94 | medium | 4181 | 268.6341524 | 3668623 | 0.43 | 4029 | d__Bacteria; p__Bacteroidota;c__Bacteroidia;o__Sphingobacteriales;f__Sphingobacteriaceae;g__Pedobacter;s__Pedobacter sp016429005 | 30d07701 | 0.008558807 | 31000 | 0.838541929 |
| concoct.30_sub | MAG_05 | 52.85 | 1.26 | medium | 1868 | 232.3076923 | 2450838 | 0.62 | 3159 | d__Bacteria; p__Actinomycetota;c__Actinomycetia;o__Propionibacteriales;f__Nocardioidaceae;g__Nocardioides;s__Nocardioides sp018831735 | 856a4314 | 0.005688744 | 14000 | 0.988546654 |
| concoct.25 | MAG_13 | 81.72 | 1.29 | medium | 4866 | 305.9399027 | 6460613 | 0.46 | 6373 | d__Bacteria; p__Bacteroidota;c__Bacteroidia;o__Chitinophagales;f__Chitinophagaceae;g__Chitinophaga;s__Chitinophaga hostae | e2eb9d22 | 0.016336241 | 103000 | 0.830741786 |
| concoct.80 | MAG_37 | 80.05 | 1.38 | medium | 7181 | 269.4011252 | 4247672 | 0.69 | 4799 | d__Bacteria; p__Pseudomonadota;c__Gammaproteobacteria;o__Burkholderiales;f__Burkholderiaceae_B;g__Variovorax;s__Variovorax sp009765735 | 0226b0c4 | 0.025659647 | 106000 | 0.989133535 |
| metabat2.47 | MAG_12 | 87.12 | 1.59 | medium | 37471 | 353.0781985 | 4686069 | 0.44 | 4041 | d__Bacteria; p__Bacteroidota;c__Bacteroidia;o__Bacteroidales;f__Bacteroidaceae;g__Bacteroides;s__Bacteroides neonati | d0d1332a | 0.123811582 | 573000 | 0.992547534 |
| concoct.67_sub | MAG_06 | 51.55 | 1.65 | medium | 1729 | 215.8222381 | 2885276 | 0.72 | 4191 | d__Bacteria; p__Actinomycetota;c__Actinomycetia;o__Propionibacteriales;f__Nocardioidaceae;g__Nocardioides;s__Nocardioides sp018831735 | 821b2eea | 0.79303208 | 2299000 | 0.995780434 |
| concoct.101_sub | MAG_08 | 64.12 | 2.63 | medium | 3769 | 266.5700991 | 3034594 | 0.41 | 3331 | d__Bacteria; p__Bacillota_A;c__Clostridia;o__Lachnospirales;f__Lachnospiraceae;g__Anaerocolumna;s__Anaerocolumna xylanovorans | 43bc704f | 0.039647577 | 117000 | 0.888557436 |
| metabat2.4_sub | MAG_33 | 86.84 | 3.75 | medium | 19306 | 323.5213102 | 5406569 | 0.67 | 5068 | d__Bacteria; p__Pseudomonadota;c__Gammaproteobacteria;o__Burkholderiales;f__Burkholderiaceae_B;g__Comamonas;s__Comamonas acidovorans | c2a411d8 | 0.887995512 | 4749000 | 0.935880562 |
| concoct.37_sub | MAG_41 | 80.77 | 3.87 | medium | 4003 | 251.9963186 | 2535214 | 0.56 | 2988 | d__Bacteria; p__Pseudomonadota;c__Gammaproteobacteria;o__Burkholderiales;f__Chromobacteriaceae;g__Aquitalea;s__Aquitalea magnusonii | f25d729c | 0.004258614 | 11000 | 0.855624522 |
| concoct.68 | MAG_32 | 100 | 5.48 | medium | 113119 | 327.5562357 | 4266263 | 0.64 | 3921 | d__Bacteria; p__Pseudomonadota;c__Gammaproteobacteria;o__Burkholderiales;f__Aquaspirillaceae;g__Microvirgula;s__Microvirgula aerodenitrificans | 5cc27572 | 0.804130889 | 3465000 | 0.960872991 |
| concoct.57 | MAG_07 | 78.52 | 6.44 | medium | 2956 | 230.6606796 | 3432409 | 0.31 | 4120 | d__Bacteria; p__Bacillota_A;c__Clostridia;o__Clostridiales;f__Clostridiaceae;g__Clostridium_J;s__Clostridium_J tunisiense | 715ce2fa | 0.003187482 | 11000 | 0.846547946 |
| concoct.56 | MAG_40 | 99.99 | 6.68 | medium | 41167 | 311.8115553 | 9589839 | 0.65 | 8931 | d__Bacteria; p__Pseudomonadota;c__Gammaproteobacteria;o__Burkholderiales;f__Burkholderiaceae;g__Cupriavidus;s__Cupriavidus basilensis | 5112c95a | 0.699700663 | 6545000 | 0.803061177 |
| maxbin.17 | MAG_03 | 98.98 | 8.55 | medium | 17934 | 291.7331334 | 3844954 | 0.69 | 4002 | d__Bacteria; p__Actinomycetota;c__Actinomycetia;o__Actinomycetales;f__Microbacteriaceae;g__Microbacterium;s__Microbacterium algeriense | a5db894d | 0.005717397 | 21000 | 0.955678585 |
| maxbin.15_sub | MAG_30 | 95.01 | 9.47 | medium | 48074 | 320.1657938 | 5832709 | 0.66 | 5537 | d__Bacteria; p__Pseudomonadota;c__Alphaproteobacteria;o__Sphingomonadales;f__Sphingomonadaceae;g__Sphingomonas;s__Sphingomonas sp017304125 | d2b5a109 | 0.290165361 | 1667000 | 0.886718425 |
| concoct.1_sub | MAG_35 | 67.99 | 9.68 | medium | 1695 | 213.4947754 | 5107203 | 0.69 | 7369 | d__Bacteria; p__Pseudomonadota;c__Gammaproteobacteria;o__Burkholderiales;f__Burkholderiaceae_B;g__Variovorax;s__Variovorax sp003019815 | dfc029c7 | 0.225864481 | 1130000 | 0.995963592 |
